# Advanced lipoprotein profile disturbances in type 1 diabetes mellitus: a focus on LDL particles

**DOI:** 10.1186/s12933-020-01099-0

**Published:** 2020-08-09

**Authors:** Antonio J. Amor, Esmeralda Castelblanco, Marta Hernández, Marga Gimenez, Minerva Granado-Casas, Jesús Blanco, Berta Soldevila, Enric Esmatjes, Ignacio Conget, Nuria Alonso, Emilio Ortega, Didac Mauricio

**Affiliations:** 1 Department of Endocrinology & Nutrition, Diabetes Unit, Hospital Clínic de Barcelona, Villarroel, 170, 08036 Barcelona, Spain; 2 Institut d’investigacions biomèdiques August Pi i Sunyer, Barcelona, Spain; 3 Department of Endocrinology & Nutrition, Hospital de la Santa Creu i Sant Pau & Institut d’Investigació Biomédica Sant Pau (IIB Sant Pau), Sant Quintí, 89, 08041 Barcelona, Spain; 4 Center for Biomedical Research on Diabetes and Associated Metabolic Diseases (CIBERDEM), Instituto de Salud Carlos III (ISCIII), Barcelona, Spain; 5 DAP-Cat Group, Unitat de Suport a la Recerca Barcelona, Fundació Institut Universitari per a la Recerca a l’Atenció Primària de Salut Jordi Gol i Gurina (IDIAPJGol), 08006 Barcelona, Spain; 6 Department of Endocrinology & Nutrition, Hospital Arnau de Vilanova & Institut d’Investigació Biomédica de Lleida (IRB Lleida), Lleida, Spain; 7 Biomedical Research Institute of Lleida & University of Lleida, Lleida, Spain; 8 Department of Endocrinology & Nutrition, Health Sciences Research Institute & University Hospital Germans Trias i Pujol, Badalona, Spain; 9 Center for Biomedical Research on Pathophysiology of Obesity and Nutrition (CIBEROBN), Instituto de Salud Carlos III (ISCIII), Barcelona, Spain

**Keywords:** Type 1 diabetes, Lipoproteins, Advanced lipoprotein profile, Nuclear magnetic resonance spectroscopy, LDL-particles

## Abstract

**Background:**

Lipoprotein disturbances have been associated with increased cardiovascular disease (CVD) risk in type 1 diabetes mellitus (T1DM). We assessed the advanced lipoprotein profile in T1DM individuals, and analysed differences with non-diabetic counterparts.

**Methods:**

This cross-sectional study involved 508 adults with T1DM and 347 controls, recruited from institutions in a Mediterranean region of Spain. Conventional and advanced (assessed by nuclear magnetic resonance [NMR] spectroscopy) lipoprotein profiles were analysed. Crude and adjusted (by age, sex, statin use, body mass index and leukocyte count) comparisons were performed.

**Results:**

The median (interquartile range) age of the study participants was 45 (38–53) years, 48.2% were men. In the T1DM group, the median diabetes duration was 23 (16–31) years, and 8.1% and 40.2% of individuals had nephropathy and retinopathy, respectively. The proportion of participants with hypertension (29.5 vs. 9.2%), and statin use (45.7% vs. 8.1%) was higher in the T1DM vs. controls (p < 0.001). The T1DM group had a better conventional (all parameters, p < 0.001) and NMR-lipid profile than the control group. Thus, T1DM individuals showed lower concentrations of atherogenic lipoproteins (VLDL-particles and LDL-particles) and higher concentrations of anti-atherogenic lipoproteins (HDL-particles) vs. controls, even after adjusting for several confounders (p < 0.001 for all). While non-diabetic women had a more favourable lipid profile than non-diabetic men, women with T1DM had a similar concentration of LDL-particles compared to men with T1DM (1231 [1125–1383] vs. 1257 [1128–1383] nmol/L, p = 0.849), and a similar concentration of small-LDL-particles to non-diabetic women (672.8 [614.2–733.9] vs. 671.2 [593.5–761.4] nmol/L, respectively; p = 0.790). Finally, T1DM individuals showed higher discrepancies between NMR-LDL-particles and conventional LDL-cholesterol than non-diabetic subjects (prevalence of LDL-cholesterol < 100 mg/dL & LDL-particles > 1000 nmol/L: 38 vs. 21.2%; p < 0.001). All these differences were largely unchanged in participants without lipid-lowering drugs (T1DM, n = 275; controls, n = 317).

**Conclusions:**

Overall, T1DM participants showed a more favourable conventional and NMR-lipid profile than controls. However, the NMR-assessment identified several lipoprotein derangements in LDL-particles among the T1DM population (higher discrepancies in NMR-LDL-particles vs. conventional LDL-cholesterol; a worse profile in T1DM women) that were overlooked in the conventional analysis. Further studies are needed to elucidate their role in the development of CVD in this population.

## Background

Cardiovascular disease (CVD) is still the leading cause of morbidity and mortality among the type 1 diabetes mellitus (T1DM) population [1]. Glycaemic control has been strongly associated with CVD risk [2]. However, even in those individuals with well-controlled T1DM (mean HbA1c < 7%), CVD death is roughly threefold higher compared with their non-diabetic counterparts [2]. Thus, other non-glycaemic CVD risk factors are involved in this accelerated atherosclerosis [3–5]. Among them, lipoprotein disturbances, especially LDL-cholesterol, are thought to be strongly related with CVD in this population. In fact, in a large cohort study, an increase of 1 mmol/L of LDL-cholesterol was associated with a 35% to 50% greater risk of overall CVD [6]. In addition, other lipid parameters such as HDL-related variables [7] and triglyceride values [8] have also been associated with CVD risk in T1DM individuals.

Advancing the understanding of the underlying lipoprotein disturbances, therefore, seems to be crucial to tackle CVD in this high-risk population beyond glycaemic control [8]. However, the information regarding the differences in the lipoprotein profile among T1DM vs. their non-diabetic counterparts is scarce and conflicting results are reported [9–11]. In addition, advanced procedures to study these lipoprotein differences in depth (i.e., nuclear magnetic resonance (NMR) spectroscopy) have been used in only a few studies, with small samples and heterogeneous results [12–15]. Furthermore, the only NMR study that recruited a large number of participants was from more than 15 years ago, which may therefore not be representative of the current T1DM population [16]. Thus, there is currently little robust evidence to draw any reliable conclusions regarding the T1DM-differential lipoprotein profile, especially in relation to contemporary cohorts and using advanced analytical tools.

Against this background, the aim of this study was to assess the advanced lipoprotein profile in T1DM individuals, and to identify differences with non-diabetic counterparts. In addition, we further analysed the differential associations between these NMR-related parameters with other common variables, such as age, gender, or anthropometric or other laboratory measurements. Finally, since LDL-cholesterol has been strongly associated with CVD, both in the general [17] and in the T1DM population [6], the differences in the conventional vs. NMR-assessed LDL-related variables were also specifically addressed.

## Methods

### Participant selection

In this cross-sectional study, 855 participants were selected from previous studies [4, 18, 19], including 508 individuals with T1DM and 347 without diabetes. This study was planned as a collaborative study that included participants from different cohorts from participating institutions from the Catalonia region of Spain that belong to the same health care organisation. All potential participants (n = 508) were identified from the electronic clinical records from the participating institutions.

The study involved three cohorts: the University Hospital Germans Trias i Pujol [UHGTiP] and University Hospital Arnau de Vilanova [UHAV] cohort; Hospital Clínic of Barcelona (HCB) cohort; and the Mollerusa cohort. Participants with T1DM were recruited from the diabetic outpatient clinics at UHGTiP-UHAV (n = 319; representing 63% of the T1DM sample), and HCB (n = 189; 37% of the T1DM sample). Participants without diabetes were from the UHGTiP-UHAV cohort (n = 192) and the Mollerussa cohort (n = 155). All the participants had available stored blood samples collected at the inclusion in each cohort.

T1DM participants included were > 18 years-old, with T1DM duration of at least 1 year, and with no evidence of previous CVD, defined as any form of clinical coronary heart disease, stroke or peripheral vascular disease (including any form of diabetic foot disease). For T1DM participants from the first two institutions (UHGTiP-UHAV), additional inclusion criteria were as follows: a) normal renal function (estimated glomerular filtration rate [eGFR] > 60 mL/min), and b) albumin-to-creatinine ratio < 300 mg/g. For T1DM from HCB, additional inclusion criteria were: a) age ≥ 40 years; b) presence of any stage of diabetic nephropathy, irrespective of the age of the subject and diabetes duration; and/or c) ≥ 10 years of duration of T1DM with at least one additional CVD risk factor (defined as either any of the following: retinopathy, family history of premature CVD in first degree relatives (defined as any CVD occurring before 55 years of age in men and before 65 years of age in women [20], active smoking habit, hypertension, low high-density lipoprotein cholesterol (HDL-cholesterol; < 40 mg/dL in males, < 45 mg/dL in females), triglycerides > 150 mg/d, the presence of severe hypoglycemia events or hypoglycemia unawareness, and women with a history of preeclampsia/eclampsia in at least one pregnancy). For the control group, participants were selected who had fasting glucose and HbA1c values below 100 mg/dl and 5.7% (39 mmol/mol), respectively, and with an available sample to assess lipoproteins. These non-diabetic subjects were free from CVD and had normal renal function as described above.

### Clinical and laboratory determinations

Age, sex, smoking habit (current, former or never smoker), and pharmacological treatment (especially focused on cardioprotective drugs) were recorded. T1DM duration was extracted from the medical records. Anthropometric data (weight, height and body mass index [BMI]) were obtained using standard methods. Waist circumference was measured to the nearest 0.5 cm using an anthropometric tape midway between the lowest rib and the iliac crest at minimal respiration. Blood pressure was registered after a few minutes of rest.

The diagnosis of diabetic retinopathy was obtained from medical records and was always verified by an ophthalmologist. Diabetic nephropathy was defined as a persistent abnormally increased creatinine-to-albumin ratio (≥ 30 mg/g) or receiving treatment with angiotensin-converting enzyme inhibitors or angiotensin receptor blockers for this reason. Obesity was defined as a BMI ≥ 30 kg/m^2^, and central obesity as a waist circumference ≥ 88 cm in women and ≥ 102 cm in men [21]. In both groups, hypertension was defined as the use of antihypertensive drugs or repeated clinical systolic blood pressure ≥ 140 mmHg or diastolic blood pressure ≥ 90 mmHg.

After at least 10–12 h of overnight fasting and without any concurrent stress the day before (minor illness, strenuous exercise, etc.), blood and first morning urine spot samples were collected and analysed in local laboratories according to standard procedures. Total cholesterol, triglycerides and HDL-cholesterol were measured directly, whereas LDL-cholesterol was estimated using the Friedewald formula. Plasma glucose concentrations were measured using the glucose oxidase method, and haemoglobin A1c (HbA1c) levels were measured using a high performance liquid chromatography method (expressed in the National Glycohemoglobin Standardization Program/Diabetes Control and Complications Trial units). Creatinine was assessed by the Jaffe method and urine albumin using an immunoturbidimetric assay. Finally, hepatic profile parameter values were determined by molecular absorption spectrometry, and inflammation markers (leukocyte count and high sensitivity C-reactive protein [hsCRP]) by flow cytometry and immunoturbidimetric assay, respectively.

Low HDL-cholesterol was defined as < 50 mg/dL in women and < 40 mg/dL in men; and high triglycerides as ≥ 150 mg/dL [21]. Non-HDL-cholesterol was calculated as total cholesterol minus HDL-cholesterol; and remnant cholesterol as total cholesterol minus HDL-cholesterol minus LDL-cholesterol [22]. The eGFR was obtained using the Chronic Kidney Disease-Epidemiology Collaboration equation (CKD-EPI) [23]. Fatty liver index (FLI) was used as a surrogate of non-alcoholic liver disease, calculated as: (e^0.953 * Loge(triglycerides) + 0.139 * BMI + 0.718 * Loge(GGT) + 0.053 * (waist circumference)−15.745^)/(1 + e ^0.953 * Loge(triglycerides) + 0.139 * BMI + 0.718 * Loge(GGT) + 0.053 * (waist circumference)−15.745^) * 100 [24]. Accordingly, hepatic steatosis was defined as a FLI > 60 [24]. Finally, in the T1DM group insulin sensitivity was estimated using the following equation that has previously been validated against euglycemic-hyperinsulinemic clamps: (log insulin sensitivity = 4.64725 − 0.02032 × waist circumference in cm − 0.00235 × triglycerides in mg/dL − 20.09779 × HbA1c in  % [25].

### Lipoprotein analysis by NMR spectroscopy (advanced lipoprotein profile)

Lipoprotein analysis of plasma samples by 2-dimensional diffusion-ordered 1H-NMR spectroscopy (DOSY) was performed as previously described [26]. This protocol evaluates the lipid concentrations (i.e., triglycerides and cholesterol), size and particle number of 3 different classes of lipoproteins (VLDL, LDL, and HDL), as well as the particle number of 9 subclasses (large, medium and small VLDL, LDL, and HDL). Briefly, the methyl signal of the plasma 2D 1H-NMR spectra was deconvoluted by using 9 Lorentzian functions to determine the lipid concentration of the large, medium and small subclasses of the main lipoprotein classes (VLDL, LDL, and HDL), and the diffusion coefficient associated with each analytical function, which is associated with the size. Finally, we combined the lipid concentration and geometric information (DC derived particle volume) in order to quantify the number of particles required to transport the measured lipid concentration of each lipoprotein subclass. Finally, weighted average VLDL, LDL, and HDL particle sizes were calculated from the subclass concentrations by summing the known diameter of each subclass multiplied by its relative percentage of subclass particle number. The mean particle diameter for the subclasses (and the estimated ranges) were as follows: large VLDL particles (VLDL-P), 75.2 nm (> 60 nm); medium VLDL-P, 52.1 nm (45–60 nm); small VLDL-P, 37.1 nm (35–45 nm); large LDL particles (LDL-P), 22.8 nm (22.5–27 nm); medium LDL-P, 20.5 nm (20–22.5 nm); small LDL-P 18.9 nm (18–20 nm); large HDL particles (HDL-P), 10.1 nm (9–13 nm); medium HDL-P, 8.7 nm (8.2–9 nm); small HDL-P, 7.8 nm (< 8.2 nm). Finally, cholesterol and triglyceride content of each lipoprotein class was also determined by using NMR-based Liposcale^®^ test [26]. All the samples were separated by centrifugation and appropriately frozen at − 80 °C from their collection, without any previous defrosting procedure before the analysis.

### Statistical analyses

Data are presented as median and 25th and 75th percentiles, mean ± standard deviation, or number (percentage) unless otherwise indicated. The normal distribution of continuous variables was assessed using the Kolmogorov–Smirnov test. Non-normally distributed variables were log transformed to reduce skewness when appropriate. Between-group differences (control vs. T1DM group) in clinical, laboratory and conventional lipid variables were evaluated using the Chi squared test for categorical variables, the Mann–Whitney test for continuous non-normally distributed variables, or the unpaired Student’s *t* test for continuous normally distributed variables.

Spearman correlation analyses were used to assess relationships between all NMR-assessed lipoproteins and age, anthropometric parameters (BMI and waist circumference), systolic blood pressure, several parameters for kidney function (eGFR and albumin-to-creatinine ratio), inflammation variables (leukocyte count and hsCRP), markers of fatty liver disease (alanine aminotransferase and FLI) and T1DM-specific variables (diabetes duration, HbA1c, insulin dose and insulin sensitivity surrogates). Differences in NMR-related variables according to study group were also assessed using parametric and non-parametric tests, as appropriate. In addition, analysis of covariance (ANCOVA) was used for adjusting these differences by age, sex and lipid-lowering drugs (model 1), and additionally by BMI and leukocyte count (model 2). The variables included in the models were selected according to the prior knowledge [27, 28], or after evaluating the significative correlations with NRM-lipoproteins found in our sample of participants. Further between-group differences in NMR-lipoprotein variables were performed only in participants without lipid-lowering drugs, or according to gender, smoking habit or chronic diabetic complications (i.e., diabetic nephropathy and retinopathy). Finally, concordance between conventional LDL-cholesterol and NRM-LDL-P according to the study group was also assessed, either as a continuous variable (Spearman correlation analysis), or using a cut-off of 100 mg/dL (for LDL-cholesterol) and 1000 nmol/L (for LDL-P). The two-sided significance level was set as p < 0.05. All statistical analyses were performed using the SPSS 20.0 statistical package (Chicago, IL).

## Results

### Participants’ characteristics

Eight hundred and fifty-five participants were included in the study. The median age of the whole sample population was 45.2 (38.0–53.0) years and 51.8% were women. The differences between those without diabetes (n = 347) vs. those with T1DM (n = 508) are shown in Table 1. All the T1DM participants were on intensified insulin therapy (either basal-bolus or continuous subcutaneous insulin infusion therapy), > 99% of them on insulin analogs. The median age, BMI, blood pressure and the proportion of men were significantly higher among T1DM individuals (p < 0.05 for all comparisons), with no significant differences in smoking habit and waist circumference. Statin use was also higher in T1DM individuals (45.7 vs. 8.1%; p < 0.001), without gender differences (men vs. women: 46.7% vs. 44.5%, p = 0.617 in the T1DM group, and 7.9% vs. 8.2%, p = 0.942 in the control group). Regarding laboratory characteristics, T1DM individuals showed higher values of some inflammation-related markers (i.e., leukocyte count; p = 0.004). As would be expected, glycaemic-related variables (fasting plasma glucose and HbA1c) were also higher in the T1DM group (p < 0.001).

**Table 1:** Differences in clinical and laboratory characteristics in study participants

	Controls	T1DM	*p* value
	(n = 347)	(n = 508)	
Clinical characteristics			
Gender (male)	151 (43.5)	261 (51.4)	0.024
Age (years)	44.0 (37.0–52.0)	46.0 (39.2–54.0)	0.005
Never smokers	160 (46.1)	248 (48.8)	0.436
Hypertension	32 (9.2)	150 (29.5)	< 0.001
SBP (mmHg)	120 (110–130)	127 (116–138)	< 0.001
DBP (mmHg)	75 (70–81)	77 (70–83)	0.044
BMI (kg/m^2^)	24.9 (22.8–27.8)	25.7 (23.1–28.4)	0.022
Obesity (BMI ≥ 30 kg/m^2^)^a^	42 (12.3)	85 (16.7)	0.074
Waist circumference (cm)	90 (82–99)	89 (82–99)	0.547
Central obesity^b^	127 (37.7)	171 (34.7)	0.376
Diabetes duration (years)	–	23.0 (16.0–31.0)	–
Diabetic nephropathy^c^	–	41 (8.1)	–
Diabetic retinopathy^d^	–	200 (40.2)	–
Statin use	28 (8.1)	232 (45.7)	< 0.001
CSII therapy	–	116 (22.8)	–
Conventional lipid profile			
Total cholesterol (mg/dL)	193 (172–219)	179 (161–202)	< 0.001
HDL-cholesterol (mg/dL)	58 (49–68)	60 (50–72)	0.035
Low HDL-cholesterol (mg/dL)^e^	45 (13.0)	50 (9.9)	0.153
LDL-cholesterol (mg/dL)	116 (96–137)	103 (86–119)	< 0.001
LDL-cholesterol < 100 mg/dL	100 (29.1)	226 (44.8)	< 0.001
LDL-cholesterol < 70 mg/dL	19 (5.5)	36 (7.1)	0.351
Triglycerides (mg/dL)	85 (63–118)	70 (54–90)	< 0.001
Triglycerides ≥ 150 mg/dL	53 (15.4)	33 (6.5)	< 0.001
Non-HDL cholesterol (mg/dL)	136 (113–160)	117 (100–136)	< 0.001
Remnant cholesterol (mg/dL)	17 (13–23)	14 (11–18)	< 0.001
Other laboratory characteristics			
Fasting plasma glucose (mg/dL)	87 (82–94)	150 (108–201)	< 0.001
Haemoglobin A1c (%)	5.4 (5.1–5.6)	7.4 (7.0–8.1)	< 0.001
Serum creatinine (mg/dL)	0.77 (0.67–0.89)	0.79 (0.68–0.91)	0.261
eGFR (CKD-EPI; ml/min/1.73 m^2^)	103 (90–111)	101 (90–110)	0.29
Alanine aminotransferase	17 (13–23)	18 (14–24)	0.021
γ-glutamyl transpeptidase	17 (12–25)	17 (12–24)	0.761
Leukocyte count (per mm^3^)	6100 (5000–7307)	6400 (5300–8012)	0.004
hsCRP (mg/L)^f^	1.13 (0.50–2.03)	1.29 (0.60–2.80)	0.088
Albumin-to-creatinine ratio	2.8 (1.4–5.0)	4.0 (2.0–7.0)	<0.001
Fatty liver index^g^	23.5 (10.1–54.8)	22.8 (10.2–47.4)	0.48
Fatty liver index > 60	67 (19.3)	71 (14.0)	0.037

Data are shown as n (percentage), mean ± standard deviation or median (Q1-Q3)

*p* values for group comparisons are reported

*BMI* Body Mass Index, *DBP* diastolic blood pressure, *CSII* continuous subcutaneous insulin insfusion, *eGFR* estimated glomerular filtration rate, *HDL* high density lipoprotein, *hsCRP* high sensitivity C-reactive protein, *LDL* low density lipoprotein, *SBP* systolic blood pressure; *T1DM* type 1 diabetes mellitus

^a^Missing values; n = 5 and n = 0

^b^Defined as ≥ 88 cm in women and ≥ 102 cm in men. Missing values, n = 10 and n = 15

^c^Missing values n = 3

^d^Missing values n = 10

^e^Defined as HDL-cholesterol < 50 in women and < 40 mg/dL in men

^f^Missing values n = 162 and n = 75

^g^Missing values n = 13 and n = 76

Baseline differences in the two cohorts of T1DM patients (UHGTiP-UHAV vs. HCB) were further assessed. There were no differences between cohorts in terms of age, main CVD risk factors, microvascular complications and statin use (p > 0.125 for all comparisons). The participants from the HCB cohort had a higher proportion of males (47.6 vs. 57.7%; p = 0.029), longer diabetes duration (mean [SD]: 20.0 [14.0–29.0] vs. 26.5 [20.6–33.4] years; p < 0.001), and some minor differences in other laboratory parameters (kidney-, liver- and inflammation-derived variables; Additional file 1: Table S1).

### Conventional and advanced lipoprotein profile in T1DM vs. controls

The conventional lipid profile according to diabetes status is shown in Table 1. Lipid concentrations were lower in T1DM individuals (p < 0.05 for all), with the exception of HDL-cholesterol (higher in T1DM; p = 0.035). No major differences were found after excluding those participants on lipid-lowering drugs (n = 33 and n = 233, for the control and T1DM groups, respectively), except for HDL-cholesterol, which was no longer statistically significant (58 [49–68] vs. 59.5 [50.7–73] mg/dL, in controls vs. T1DM, respectively; p = 0.065).

The NMR advanced lipoprotein profile showed further differences (Table 2). Thus, VLDL-P and LDL-P lipid content (both cholesterol and triglycerides), and total particle number and subclasses were significantly lower in the T1DM group (all p < 0.01). Regarding HDL, most of the related variables were higher in the T1DM group (i.e., the particle number and subclasses, and lipid content; p < 0.01 for all comparisons). Consequently, several atherogenic lipid markers were also found to be lower in the T1DM group (p < 0.001). VLDL-P and LDL-P size was similar, and HDL-P size higher, in the T1DM group. No differences were observed after adjusting for age, gender, statin use, BMI or leukocyte count (Table 2). These differences in the NMR lipoprotein profile were also assessed only in the participants without lipid-lowering treatment (for the characteristics, see Additional file 2: Table S2). Overall, all the changes described above in the whole sample of individuals regarding T1DM vs. controls remained unchanged in the participants without lipid-lowering treatment (Table 3).

**Table 2:** NMR-assessed advanced lipoprotein profile in control and T1DM groups

NMR variable	Controls	T1DM	p	p^*^	p^†^
	(n = 347)	(n = 508)			
VLDL-P number (nmol/L)					
Total	36.5 (25.5–53.3)	29.1 (22.8–37.7)	< 0.001	< 0.001	< 0.001
Large	0.93 (0.71–1.24)	0.82 (0.63–1.04)	< 0.001	< 0.001	< 0.001
Medium	3.92 (2.62–5.50)	2.80 (1.88–4.28)	< 0.001	< 0.001	< 0.001
Small	31.4 (22.1–47.0)	25.4 (20.1–32.5)	< 0.001	< 0.001	< 0.001
Ratio large/total	0.025 (0.023–0.027)	0.027 (0.025–0.030)	< 0.001	< 0.001	< 0.001
VLDL-P composition (mg/dL)					
VLDL-C	9.94 (5.44–16.11)	7.78 (4.42–11.80)	0.001	< 0.001	< 0.001
VLDL-TG	52.8 (36.5–75.3)	41.0 (32.2–53.5)	< 0.001	< 0.001	< 0.001
Ratio VLDL-C/VLDL-TG	0.17 (0.13–0.21)	0.19 (0.13–0.23)	0.003	0.056	0.075
VLDL-P size (nm)	42.1 (42.0–42.3)	42.1 (41.9–42.3)	0.348	0.502	0.469
LDL-P number (nmol/L)					
Total	1356.2 (1159.3–1567.7)	1244.6 (1127.7–1382.6)	< 0.001	< 0.001	< 0.001
Large	191.9 (167.6–217.6)	175.7 (159.9–195.2)	< 0.001	< 0.001	< 0.001
Medium	437.6 (344.6–531.3)	373.4 (315.5–461.0)	< 0.001	< 0.001	< 0.001
Small	708.6 (623.5–827.3)	684.1 (622.0–751.7)	0.001	< 0.001	< 0.001
Ratio small/total	0.53 (0.49–0.58)	0.55 (0.51–0.59)	< 0.001	0.094	0.098
LDL-P composition (mg/dL)					
LDL-C	132.9 (113.5–154.2)	122.4 (110.1–137.0)	< 0.001	< 0.001	< 0.001
LDL-TG	16.6 (13.4–20.4)	14.6 (12.5–17.6)	< 0.001	< 0.001	< 0.001
Ratio LDL-C/LDL-TG	8.20 (7.25–9.13)	8.25 (7.35–9.60)	0.042	0.003	0.003
LDL-P size (nm)	21.0 ± 0.27	21.0 ± 0.27	0.35	0.163	0.142
HDL-P number (μmol/L)					
Total	29.0 (26.0–33.2)	31.8 (29.2–36.0)	< 0.001	< 0.001	< 0.001
Large	0.26 (0.23–0.30)	0.27 (0.25–0.31)	0.002	< 0.001	< 0.001
Medium	9.12 (7.90–10.60)	10.47 (9.19–12.11)	< 0.001	< 0.001	< 0.001
Small	19.9 (17.4–22.8)	20.9 (18.5–23.8)	0.001	0.009	0.005
Ratio small/total	0.67 (0.64–0.71)	0.66 (0.63–0.69)	< 0.001	< 0.001	< 0.001
HDL-P composition (mg/dL)					
HDL-C	56.2 (49.2–65.5)	63.0 (54.9–73.3)	< 0.001	< 0.001	< 0.001
HDL-TG	12.7 (10.3–15.7)	14.7 (12.2–17.7)	< 0.001	< 0.001	< 0.001
Ratio HDL-C/HDL-TG	4.49 (3.61–5.52)	4.32 (3.58–5.31)	0.101	0.315	0.215
HDL-P size (nm)	8.21 ± 0.06	8.25 ± 0.07	< 0.001	< 0.001	< 0.001
Other atherogenic variables					
Non-HDL-P (nmol/L)	1377.2 (1170.7–1578.5)	1239.8 (1128.6–1382.4)	< 0.001	< 0.001	< 0.001
Ratio LDL-P/HDL-P	46.1 (37.2–56.7)	39.4 (33.3–45.5)	< 0.001	< 0.001	< 0.001
Ratio total-P/HDL-P	47.7 (38.3–58.4)	40.4 (34.0–46.7)	< 0.001	< 0.001	< 0.001

Data are shown as median (Q1–Q3) or mean ± standard deviation

*p-value adjusted for age, sex, and lipid-lowering medications

^†^p-value adjusted for age, sex, lipid-lowering medications, BMI and leukocyte count

*HDL* high-density lipoprotein, *HDL-C* cholesterol content in HDL, *HDL-P* HDL particles, *HDL-TG* triglyceride content in HDL, *LDL* low-density lipoprotein, *LDL-C* cholesterol content in LDL, *LDL-P* LDL particles, *LDL-TG* triglyceride content in LDL, *NMR* nuclear magnetic resonance, *T1DM* type 1 diabetes mellitus, *VLDL* very low-density lipoprotein, *VLDL-C* cholesterol content in VLDL, *VLDL-P* VLDL particles, *VLDL-TG* triglyceride content in VLDL

**Table 3:** NMR-assessed advanced lipoprotein profile in participants without lipid-lowering drugs

NMR variable	Controls	T1DM	p	p*	p^†^
	(n = 317)	(n = 275)			
VLDL-P number (nmol/L)					
Total	36.0 (25.3–52.4)	27.6 (21.6–36.0)	< 0.001	< 0.001	< 0.001
Large	0.93 (0.69–1.22)	0.76 (0.60–1.03)	< 0.001	< 0.001	< 0.001
Medium	3.88 (2.53–5.43)	2.68 (1.87–4.18)	< 0.001	< 0.001	< 0.001
Small	31.3 (21.9–45.4)	23.8 (19.1–30.7)	< 0.001	< 0.001	< 0.001
Ratio large/total	0.025 (0.023–0.028)	0.027 (0.025–0.030)	< 0.001	< 0.001	< 0.001
VLDL-P composition (mg/dL)					
VLDL-C	9.85 (5.37–15.76)	7.22 (4.16–11.70)	0.002	0.001	0.001
VLDL-TG	52.0 (36.4–73.9)	38.2 (30.8–50.3)	< 0.001	< 0.001	< 0.001
Ratio VLDL-C/VLDL-TG	0.17 (0.13–0.21)	0.19 (0.13–0.23)	0.007	0.047	0.054
VLDL-P size (nm)	42.1 (42.0–42.3)	42.1 (41.9–42.3)	0.877	0.352	0.351
LDL-P number (nmol/L)					
Total	1342.6 (1159.1–1551.7)	1276.8 (1151.7–1406.0)	< 0.001	0.001	< 0.001
Large	191.9 (167.8–216.5)	181.7 (167.1–203.0)	0.002	0.015	0.01
Medium	433.6 (345.2–522.9)	391.8 (331.6–471.6)	< 0.001	0.006	0.003
Small	707.2 (618.9–815.2)	686.9 (620.7–755.0)	0.035	0.013	0.004
Ratio small/total	0.53 (0.49–0.58)	0.54 (0.51–0.57)	0.058	0.134	0.138
LDL-P composition (mg/dL)					
LDL-C	132.5 (113.4–152.6)	126.0 (114.4–138.6)	0.004	0.014	0.006
LDL-TG	16.2 (13.3–20.1)	14.4 (12.4–17.8)	< 0.001	< 0.001	< 0.001
Ratio LDL-C/LDL-TG	8.30 (7.29–9.21)	8.61 (7.54–9.95)	0.003	0.006	0.007
LDL-P size (nm)	21.0 ± 0.27	21.1 ± 0.24	0.388	0.185	0.158
HDL-P number (μmol/L)					
Total	28.7 (25.7–33.02)	31.1 (27.5–35.6)	< 0.001	< 0.001	< 0.001
Large	0.26 (0.23–0.30)	0.28 (0.25–0.31)	0.001	< 0.001	< 0.001
Medium	9.04 (7.88–10.62)	10.43 (9.15–12.24)	< 0.001	< 0.001	< 0.001
Small	19.7 (17.1–22.7)	20.4 (17.9–23.2)	0.079	0.018	0.012
Ratio small/total	0.67 (0.64–0.71)	0.65 (0.62–0.68)	< 0.001	< 0.001	< 0.001
HDL-P composition (mg/dL)					
HDL-C	56.1 (49.0–65.5)	63.0 (54.6–73.8)	< 0.001	< 0.001	< 0.001
HDL-TG	12.5 (10.1–15.6)	13.7 (11.4–16.2)	< 0.001	< 0.001	< 0.001
Ratio HDL-C/HDL-TG	4.58 (3.63–5.59)	4.64 (3.80–5.68)	0.494	0.427	0.364
HDL-P size (nm)	8.21 ± 0.06	8.25 ± 0.07	< 0.001	< 0.001	< 0.001
Other atherogenic variables					
Non-HDL-P (nmol/L)	1369.5 (1169.6–1574.9)	1274.6 (1154.1–1406.9)	< 0.001	< 0.001	< 0.001
Ratio LDL-P/HDL-P	47.4 (38.5–58.3)	41.9 (35.5–48.5)	< 0.001	< 0.001	< 0.001
Ratio total-P/HDL-P	45.9 (37.4–56.4)	41.1 (34.6–47.1)	< 0.001	< 0.001	< 0.001

Data are shown as median (Q1–Q3) or mean ± standard deviation

*p-value adjusted for age and sex

^†^p-value adjusted for age, sex, BMI and leukocyte count

*HDL* high-density lipoprotein, *HDL-C* cholesterol content in HDL, *HDL-P* HDL particles, *HDL-TG* triglyceride content in HDL, *LDL* low-density lipoprotein, *LDL-C* cholesterol content in LDL, *LDL-P* LDL particles, *LDL-TG* triglyceride content in LDL, *NMR* nuclear magnetic resonance, *T1DM* type 1 diabetes mellitus, *VLDL* very low-density lipoprotein, *VLDL-C* cholesterol content in VLDL, *VLDL-P* VLDL particles, *VLDL-TG* triglyceride content in VLDL

As an additional analysis, the NMR-variables between the two T1DM cohorts (UHGTiP-UHAV and HCB cohorts) were also compared. Compared to the HCB cohort group, the UHGTiP-UHAV cohort group showed lower concentrations of VLDL-P and higher concentrations of HDL-P (p < 0.01 for all), but similar amounts of total LDL-P and non-HDL-P (p > 0.800 for both); this is in accordance with the conventional lipid profile (Additional file 1: Table S1). Sex-adjusted models blunted some of these differences, especially in HDL-related variables. Both T1DM cohorts compared separately to the control group showed similar results to that observed in the whole T1DM group (Additional file 3: Table S3).

### Sex and age differences in the NMR advanced lipoprotein profile

With regard to sex comparisons within the same-group (either control or T1DM), women showed lower values of VLDL and higher values of HDL-related variables (all p < 0.05, Table 4). LDL-P was significantly lower in women vs. men in the control group (1413 [1204–1624] vs. 1315 [1136–1506] nmol/L; p = 0.003], but there were no such significant differences in the T1DM group (1257 [1128–1383] vs. 1231 [1125–1383]; p = 0.849). Similarly, non-HDL-P was significantly lower in women vs. men in the control group (p < 0.001), but not in the T1DM group (p = 0.342). In same-sex comparisons across groups, all the main variables were lower in men or women from the T1DM group (all p < 0.001), except for small LDL-P in women (671.2 [593.5–761.4] vs. 672.8 [614.2–733.9] nmol/L, for the control and T1DM groups, respectively; p = 0.790). No major changes were found in those participants without lipid-lowering drugs (Additional file 4: Table S4).

**Table 4:** NMR-assessed advanced lipoprotein profile in control and T1DM groups according to gender

NMR variable	Control (n = 347)			T1DM (n = 508)			Men (control vs. T1DM)	Women (control vs. T1DM)
	Men	Women	p	Men	Women	p		
VLDL-P number (nmol/L)								
Total	44.2 (31.5–67.8)	32.0 (23.8–45.0)	< 0.001	31.4 (25.0–43.6)	25.9 (21.2–32.6)	< 0.001	< 0.001	< 0.001
Large	1.06 (0.81–1.51)	0.86 (0.60–1.100)	< 0.001	0.93 (0.73–1.24)	0.71 (0.56–0.94)	< 0.001	0.001	< 0.001
Medium	4.45 (2.94–6.32)	3.36 (2.36–4.94)	< 0.001	3.29 (2.32–4.83)	2.51 (1.68–3.70)	< 0.001	< 0.001	< 0.001
Small	37.5 (27.7–59.6)	27.7 (20.2–39.4)	< 0.001	27.3 (22.0–37.6)	22.8 (18.8–28.1)	< 0.001	< 0.001	< 0.001
Ratio large/total	0.024 (0.023–0.027)	0.026 (0.023–0.028)	0.012	0.028 (0.025–0.030)	0.027 (0.025–0.029)	0.002	< 0.001	< 0.001
VLDL-P composition (mg/dL)								
VLDL-C	11.30 (6.54–19.40)	7.64 (4.39–12.85)	< 0.001	8.99 (5.64–14.60)	6.64 (3.68–9.41)	< 0.001	0.006	0.003
VLDL-TG	62.1 (44.0–94.0)	45.8 (34.0–63.2)	< 0.001	43.9 (35.2–62.4)	36.7 (29.2–45.2)	< 0.001	< 0.001	< 0.001
Ratio VLDL-C/VLDL-TG	0.17 (0.13–0.21)	0.17 (0.12–0.21)	0.451	0.20 (0.15–0.24)	0.17 (0.12–0.22)	0.001	0.001	0.429
VLDL-P size (nm)	42.08 (41.96–42.24)	42.15 (41.98–42.32)	0.041	42.14 (41.95–42.33)	42.10 (41.87–42.25)	0.03	0.135	0.008
LDL-P number (nmol/L)								
Total	1413 (1204–1624)	1315 (1137–1506)	0.003	1257 (1128–1383)	1231 (1125–1383)	0.849	< 0.001	0.008
Large	191.4 (166.2–218.0)	192.2 (169.2–215.0)	0.957	174.8 (159.1–191.5)	176.2 (160.1–200.9)	0.419	< 0.001	< 0.001
Medium	432.1 (323.7–534.7)	441.1 (351.3–530.3)	0.475	364.8 (304.9–444.2)	387.0 (324.0–489.2)	0.008	< 0.001	< 0.001
Small	778.7 (692.9–878.4)	671.2 (593.5–761.4)	< 0.001	693.4 (635.7–766.2)	672.8 (614.2–733.9)	0.002	< 0.001	0.79
Ratio small/total	0.56 (0.52–0.60)	0.51 (0.48–0.55)	< 0.001	0.56 (0.52–0.60)	0.54 (0.49–0.58)	< 0.001	0.607	< 0.001
LDL-P composition (mg/dL)								
LDL-C	137.0 (116.9–157.2)	130.7 (111.3–149.6)	0.05	123.2 (110.0–137.2)	121.2 (110.2–136.9)	0.798	< 0.001	0.005
LDL-TG	16.5 (13.0–20.1)	16.8 (13.7–20.6)	0.656	14.1 (12.2–16.9)	15.1 (12.7–18.2)	0.013	< 0.001	0.002
Ratio LDL-C/LDL-TG	8.48 (7.55–9.56)	7.96 (7.00–8.92)	0.003	8.62 (7.61–9.79)	8.07 (7.14–9.31)	< 0.001	0.304	0.169
LDL-P size (nm)	20.9 ± 0.26	21.1 ± 0.24	< 0.001	21.0 ± 0.27	21.1 ± 0.27	< 0.001	0.035	0.01
HDL-P number (μmol/L)								
Total	27.3 (24.3–29.9)	31.2 (27.8–35.1)	< 0.001	29.6 (26.5–33.6)	34.2 (30.8–37.6)	< 0.001	< 0.001	< 0.001
Large	0.25 (0.23–0.29)	0.28 (0.25–0.31)	< 0.001	0.26 (0.24–0.29)	0.29 (0.26–0.33)	< 0.001	0.015	0.001
Medium	8.06 (7.18–9.23)	9.99 (8.81–11.48)	< 0.001	9.67 (8.58–10.82)	11.8 (10.0–13.7)	< 0.001	< 0.001	< 0.001
Small	18.8 (16.2–21.0)	21.0 (1.83–23.7)	< 0.001	19.9 (17.2–22.7)	22.0 (19.5–24.6)	< 0.001	0.005	0.004
Ratio small/total	0.69 (0.65–0.71)	0.67 (0.64–0.70)	< 0.001	0.67 (0.64–0.69)	0.65 (0.62–0.67)	< 0.001	< 0.001	< 0.001
HDL-P composition (mg/dL)								
HDL-C	51.4 (45.8–58.1)	59.8 (53.0–69.7)	< 0.001	58.3 (50.9–65.5)	69.0 (61.6–79.6)	< 0.001	< 0.001	< 0.001
HDL-TG	11.31 (9.05–14.03)	13.6 (11.4–16.6)	< 0.001	13.9 (11.5–17.1)	15.4 (13.2–18.8)	< 0.001	< 0.001	< 0.001
Ratio HDL-C/HDL-TG	4.59 (3.64–5.66)	4.40 (3.58–5.42)	0.331	4.22 (3.43–5.14)	4.43 (3.71–5.41)	0.022	0.013	0.782
HDL-P size (nm)	8.20 ± 0.06	8.22 ± 0.05	< 0.001	8.24 ± 0.07	8.26 ± 0.06	0.002	< 0.001	< 0.001
Other atherogenic variables								
Non-HDL-P (nmol/L)	1446 (1224–1639)	1316 (1130–1513)	< 0.001	1260 (1133–1395)	1225 (1123–1369)	0.342	< 0.001	0.003
Ratio LDL-P/HDL-P	52.6 (44.0–63.3)	41.1 (34.2–50.8)	< 0.001	41.7 (35.7–49.4)	36.0 (31.6–42.8)	< 0.001	< 0.001	< 0.001
Ratio total-P/HDL-P	54.0 (45.5–65.5)	42.1 (35.3–52.0)	< 0.001	43.0 (36.6–51.2)	36.6 (32.4–43.7)	< 0.001	< 0.001	< 0.001

Data are shown as median (Q1–Q3) or mean ± standard deviation

No differences in the statin use between gender (p = 0.942 and p = 0.617, for the control and T1DM group, respectively)

*HDL* high-density lipoprotein, *HDL-C* cholesterol content in HDL, *HDL-P* HDL particles, *HDL-TG* triglyceride content in HDL, *LDL* low-density lipoprotein, *LDL-C* cholesterol content in LDL, *LDL-P* LDL particles, *LDL-TG* triglyceride content in LDL, *NMR* nuclear magnetic resonance, *T1DM* type 1 diabetes mellitus, *VLDL* very low-density lipoprotein, *VLDL-C* cholesterol content in VLDL, *VLDL-P* VLDL particles, *VLDL-TG* triglyceride content in VLDL

**Fig. 1:**
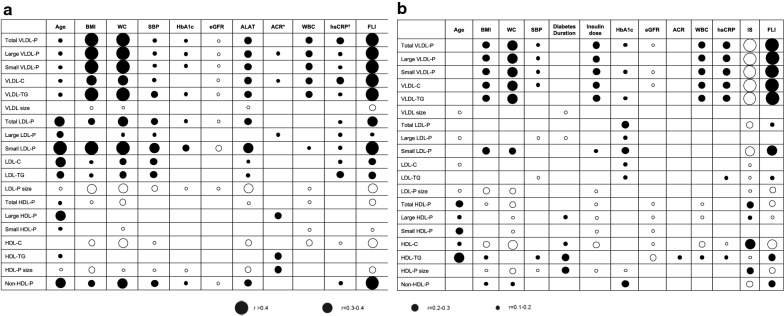
Associations between NMR-assessed advanced lipoprotein profile and clinical and laboratory parameters. **a** Control Group; **b** T1DM group. Solid and open circles indicate positive and negative relationships, respectively. * Available value in n = 191 controls. ^†^ Available value in n = 185 controls. ACR: albumin-to-creatinine ratio; ALAT: alanine aminotransferase: BMI: body mass index; eGFR: estimated glomerular filtration rate; FLI: fatty liver index; FPG: fasting plasma glucose; HDL: high-density lipoprotein; HDL-C: cholesterol content in HDL; HDL-P: HDL particles; HDL-TG: triglyceride content in HDL; hsCRP: high sensitivity C-reactive protein; IS: insulin sensitivity; LDL: low-density lipoprotein; LDL-C: cholesterol content in LDL; LDL-P: LDL particles; LDL-TG: triglyceride content in LDL; NMR: nuclear magnetic resonance; SBP: systolic blood pressure; VLDL: very low-density lipoprotein; VLDL-C: cholesterol content in VLDL; VLDL-P: VLDL particles; VLDL-TG: triglyceride content in VLDL; WBC: white blood cells; WC: waist circumference

Overall, in both groups there was a direct relationship between age and HDL-related variables, whereas stronger and direct relationships in LDL-related parameters were only observed in the control group (Fig. 1). After dividing the sample into age quintiles, different patterns were found in the control vs. T1DM groups according to sex (Additional file 5: Fig. S1). In statin-adjusted models, VLDL-P concentrations (and their lipid content) were lower, and HDL-P concentrations (and their cholesterol content) were higher in women vs. men in both groups, and also in the T1DM group vs. the control group, in almost all the age ranges. LDL-related particles (LDL-P and LDL-C) showed a different pattern. In the control group, men had a marked increase in the levels of LDL-related variables between the ages of 36 to 48 years (being significantly higher than women), with a stepped decrease after this age range. Women in the control group, however, showed a stepped increase over all the age ranges (p < 0.01), with a similar number of particles and cholesterol content than men from the age of 48 years. In the T1DM group there were blunted changes in these variables across the age ranges, as well as lower between-gender differences. In fact, LDL-P and LDL-C was only higher in men (vs. women) in the age range of 36–42 years, while in participants > 56 years both parameters were significantly higher in T1DM women. Non-HDL-P followed the same pattern as LDL-related parameters, being also higher in women (vs. men) only in T1DM participants over 56 years-old.

### Relationships between the advanced lipoprotein profile and other variables

The relationships between the NMR advanced lipoprotein profile according to several traits are shown in Fig. 1. In both the control and T1DM groups, BMI, waist circumference, and FLI were the variables most strongly associated with NMR variables, directly with VLDL-related variables, small LDL-P and non-HDL-P, and inversely with HDL-related parameters. Weaker associations were found with inflammation-related markers (i.e. leukocyte count and hsCRP), especially with the VLDL-related variables (direct). Regarding T1DM-specific variables, there were no associations with diabetic nephropathy measurements (Fig. 1b) or retinopathy (data not shown), although both diabetes duration (r = 0.2–0.3 for HDL-TG or HDL size; Fig. 1b) and glycaemic control (r = 0.2–0.3 for total LDL-P and small LDL-P; Fig. 1b) were associated with some NMR-lipoproteins. In fact, the stratification of T1DM participants according to HbA1c concentrations (< 7, 7–8.5 and > 8.5%) revealed a stepped increase in the atherogenic particles (VLDL-P, LDL-P and non-HDL-P; p < 0.05 for all), and a decrease in HDL size (p < 0.01; Additional file 6: Table S5) with increasing HbA1c values. Other T1DM-specific variables also showed strong correlations with lipoproteins, especially insulin sensitivity markers (r > 0.4 for VLDL-related variables; r = 0.2–0.3 for some HDL-variables; Fig. 1b). No major differences were found in participants without lipid-lowering drugs (Additional file 7: Fig. S2). Finally, while no differences were found in the control group, the group with T1DM individuals with an active smoking habit showed significantly lower levels of HDL-P (32.1 [28.9–36.4] vs. 30.6 [26.3–34.8] μmol/L; p = 0.037 in multivariate-adjusted models; data not shown).

### Conventional vs. NMR-derived LDL-related parameters

**Fig. 2:**
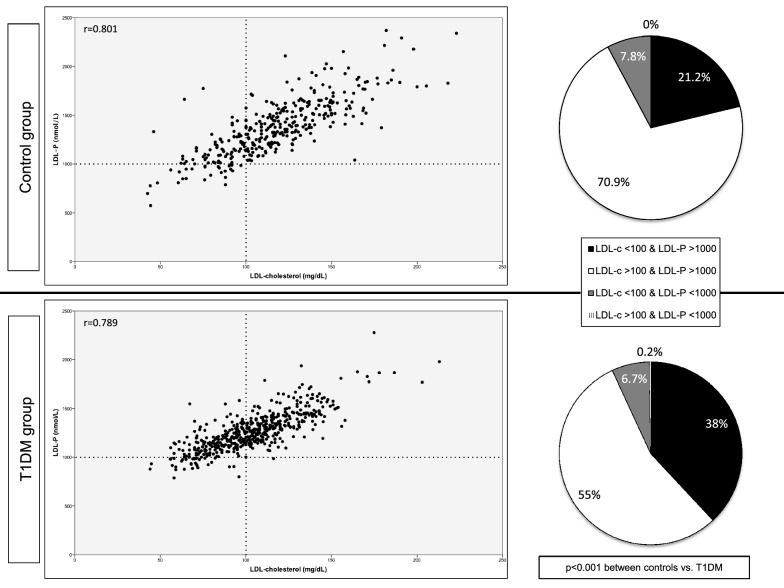
Scatterplots between LDL-cholesterol measured by conventional methods and LDL-P measured by NMR spectroscopy and concordance according to LDL-cholesterol < or > 100 mg/dL and LDL-P < or > 1000 nmol/l in the control (upper) and T1DM group (down)

LDL-cholesterol assessed by conventional methods and LDL-P measured by NMR spectroscopy were strongly correlated in both groups (r = 0.801 and r = 0.789, for control and T1DM groups, respectively; Fig. 2). However, a higher number of LDL particles per mg/dl of LDL-cholesterol (ratio LDL-P/LDL-cholesterol) was found in the T1DM group (11.71 [10.61–13.07] vs. 12.19 [11.21–13.65], for the control and T1DM groups, respectively; p < 0.001). In fact, when we assessed the percentage of participants with concordance in LDL-cholesterol and LDL-P levels (< or > 100 mg/dL and < or > 1000 nmol/L, respectively), almost twice the proportion of participants with T1DM and LDL-cholesterol < 100 mg/dL had LDL-P > 1000 nmol/L (21.2% for the control group vs. 38% for T1DM group; p < 0.001; Fig. 2). Significant differences still remained after excluding those participants on lipid-lowering drugs (20.5% vs. 29.1%; p = 0.011). Interestingly, although the proportion of participants with LDL-cholesterol < 100 mg/dl was significantly higher in the T1DM group (29.1% vs. 44.8%; p < 0.001; Table 1), no difference was observed in the proportion with LDL-P < 1000 nmol/L (7.8 vs. 7.0%, for the control and T1DM group, respectively; p = 0.622).

## Discussion

In this large sample of individuals with T1DM, a better overall advanced lipoprotein profile was observed compared to non-diabetic counterparts, characterised by a decrease in the total amount of atherogenic particles (i.e., VLDL-P and LDL-P) and an increase in antiatherogenic ones (i.e., HDL-P). However, a differential pattern in LDL-related variables was shown in this population, with a higher number of LDL-P per each mg/dl of conventional LDL-cholesterol, and a worse profile among T1DM women (similar small LDL-P than women without diabetes; no differences in total LDL-P compared with men with T1DM). To the best of our knowledge, this is so far the largest study assessing the differences in NMR-assessed lipoprotein profile in a broad range of contemporary patients with T1DM.

### Differences in NMR advanced lipoprotein profile according to T1DM status and gender

In our sample of T1DM individuals from a Mediterranean region of Spain, a decrease in the total number of atherogenic lipoproteins and an increase in the antiatherogenic ones was shown vs. non-diabetic counterparts (p < 0.001 for all comparisons, Table 2). However, previous studies have reported conflicting results, either using conventional biochemical analysis [9], liquid chromatography [10, 11], proteomic analysis [29] or RMN-advanced procedures [12–16]. Overall, although the majority of the studies showed a better overall lipid profile among T1DM individuals [9–13, 16], two independent studies suggested that this population could have proatherogenic changes in the lipoproteins [14, 15]. Specifically, higher amounts of total LDL-P and small HDL-P were found in adolescents [15], and an enrichment in triglyceride content (vs. cholesterol) in all the lipoprotein classes was found in adults with T1DM [14]. In contrast, no sign of the subtle proatherogenic changes suggested in the previous studies [14, 15] was found in our sample, as the triglyceride content of the main lipoprotein classes was not increased (or even decreased in the case of LDL-P), and the mean size of the HDL-P was significantly higher in the T1DM group. Some differences between the previous studies and ours should be acknowledged, such as the inclusion of an older cohort that may not be representative of the current management of T1DM (either in glucose or CVD risk factor control) [9, 16], the limited sample size [10–14], or the strict inclusion criteria of the patients [15]. Furthermore, underlying dietetic factors should also be taken into account. In fact, the Mediterranean diet has been associated with an improved lipoprotein profile in previous studies [30]. Since the T1DM population from our geographical area has been shown to have a greater adherence this type of diet [31], this could have influenced our findings in T1DM vs. controls.

The presence of T1DM in females seems to be associated with a greater proportional CVD risk than in men. Thus, a recent meta-analysis showed that women with T1DM had roughly a two-fold greater excess risk of fatal and non-fatal CVD compared with T1DM men [32]. Although some gender-specific pregnancy complications may help to explain this excess risk [3], lipid factors may also be involved. In fact, in our contemporary sample, T1DM women showed a worse NMR profile, characterised by a similar amount of small LDL-P than women without T1DM; and the same LDL-P and total atherogenic particles (non-HDL-P) than men with T1DM (Table 4). In addition, our data further showed that the between-gender differences in LDL-P and non-HDL-P were less pronounced in the T1DM group vs. the control group across all the age ranges of the individuals, and even T1DM women had significantly higher levels of these lipoproteins from the age of 56 years (Additional file 5: Fig. S1). Statin treatment was 5 to sixfold higher in the T1DM group vs. the control group and this could have influenced the between-group relationships, however the between-gender differences were less prone to being affected by this variable, since statin use was similar in men and women (Table 4). Our data agree with some older studies, which have also pointed out that the better lipoprotein profile observed in the T1DM population (vs. control) was usually blunted in women [10, 16], with similar between-gender levels of total and LDL-cholesterol in T1DM patients [33].

### NMR advanced lipoprotein profile and insulin resistance surrogates

Insulin resistance is strongly associated with several lipoprotein profile derangements [34]. Previous NMR studies performed in non-diabetic and type 2 diabetes mellitus patients have shown that either markers of adiposity or several non-invasive scores of fatty liver disease (both closely related with insulin resistance) were directly associated with VLDL-related variables, and inversely with HDL-related variables [27, 28]. In our study, the adiposity marker most closely associated with insulin resistance (i.e. waist circumference) and the most widely used fatty liver disease score (i.e. FLI) were the variables most strongly associated with the NMR-advanced lipoprotein profile, both in T1DM or control individuals (Fig. 1): direct correlations were observed with triglyceride-rich lipoproteins and small LDL-P, and inverse correlations with HDL-P. Further, some T1DM-specific surrogates of insulin sensitivity were also associated with these NMR-parameters. Preliminary previous data in T1DM has also shown similar associations between insulin resistance markers and a more proatherogenic lipoprotein profile [10, 33, 35]; but information regarding NMR-assessed analysis has been lacking. Some of these NMR-variables observed in our study, previously associated with CVD and mortality in T1DM populations [7, 36, 37], could also mediate the well-known relationships between insulin resistance and vascular complications in T1DM [38–40].

### LDL-related parameters

HbA1c showed a strong and direct association with LDL-related variables in our T1DM sample (Fig. 1 and Additional file 6: Table S5), in accordance with previous studies [33, 35, 41–43]. Specifically, two recent studies performed in children and young adults with T1DM also showed a direct association between glycaemic control and overall dyslipidaemia [42], and LDL-cholesterol (this effect was further modified by proprotein convertase subtilisin/kexin 9 levels) [43]. As is well known, LDL-cholesterol has been pointed out as a causal factor in the pathogenesis of atherosclerotic CVD in the general population [44]. Although the factors involved among the T1DM population are more complex [3–5], LDL-related variables remain as one of the most important lipid parameters for CVD risk [6, 45]. In fact, this variable was strongly associated with coronary atherosclerosis in a study assessing long-standing T1DM participants [46]. Thus, increasing the knowledge of the variables associated with LDL-cholesterol levels in this population seems to be crucial for CVD risk assessment. Hence, this relationship between HbA1c and LDL-related variables could also be involved in mediating the well-known association between poor glycaemic control and increased CVD among the T1DM population [47].

Discrepancies between conventional LDL-cholesterol and NMR-LDL parameters were found in T1DM individuals. In our sample, the T1DM group showed higher NRM-LDL-P vs. conventional LDL-cholesterol compared to controls (38% vs. 21.1%; Fig. 2). Some NMR-derived LDL variables seem to confer additional benefits in CVD risk assessment, beyond conventional measurements of LDL-cholesterol [48]. Prospective data in the general population also showed that the individuals with discordant values (i.e., those with higher LDL-P in relation to conventional LDL-cholesterol values) were those with a higher incidence of future CVD events [49, 50]. Thus, the increased proportion of T1DM individuals with discordant values between the NMR vs. the conventional analysis could counteract the apparent “normal” conventional LDL-cholesterol levels among the T1DM population, and may partially explain why LDL-cholesterol could be more atherogenic in T1DM [51]. In this sense, the lowered targets of LDL-cholesterol in T1DM recently released by some societies [17] could help to overcome this fact.

### Strengths and limitations

Several strengths and limitations of our study deserve additional comments. Among the strengths, we included a large number of T1DM participants and controls with a broad age span. In this regard, we could show the evolution of the advanced lipoprotein profile in different age ranges, for which there is a paucity of data to date. In addition, the contemporary T1DM individuals included, reflecting the current state-of-art in glucose and CVD risk factor management, is a more representative image of the actual T1DM population than the older studies assessing the same topic [9, 16]. Finally, we used the most robust method available to study lipoprotein metabolism, showing many advantages compared with older tests [52]. Several limitations should also be acknowledged. First, due to the unmatched design, some of the variables closely related to the lipoprotein profile were different between the T1DM group and the control group. In this regard, statin treatment was significantly higher in T1DM individuals, which could have influenced our results. However, most of our results were adjusted for statin treatment, and a sensitivity analysis performed in patients without lipid-lowering treatment led to similar results (either in the differences between T1DM vs. controls, between-gender differences, or in the correlations between NMR-lipoproteins and other clinical and laboratory variables). In addition, some studies have suggested that individuals with well-controlled T1DM could have lower LDL-cholesterol levels than their non-diabetic counterparts due to decreases in VLDL production or increases in LDL catabolism [53]. Since our sample of T1DM participants was fairly controlled (median HbA1c of 7.4%), this fact could partially explain our main findings. Second, the inclusion of T1DM participants from cohorts with different inclusion criteria could induce some bias in the selection of the sample. However, since a broad range of participants were included, it could make our results more generalizable. Furthermore, because the differences in NRM advanced lipoprotein profile between T1DM vs. control groups were consistent regardless of the study cohort, this further supports our main findings. Third, because virtually all of our T1DM participants were on insulin analogs, we could not assess whether the use of this type of insulin was associated with changes in the advanced lipoprotein profile. Since preliminary reports have shown a borderline lower CVD mortality among T1DM users of this type of therapy [54], it would be interesting to study whether this could be mediated through an improved lipoprotein profile. Fourth, the exclusion of participants without established CVD could lead to a selection bias, especially for the T1DM group. Thus, the extrapolation of our results to other T1DM patients at higher risk should be made with caution. Finally, due to our cross-sectional design, causality cannot be drawn in the associations between advanced lipid profile and the other variables.

## Conclusions

In our contemporary sample of Mediterranean individuals with T1DM without CVD, a better overall advanced lipoprotein profile vs. non-diabetic participants was observed. However, although total LDL-P was significantly lower, T1DM individuals showed a greater prevalence of discordance with conventional LDL-cholesterol. Furthermore, a differentiated pattern was observed according to gender, with less pronounced between-group differences in women with T1DM (vs. T1DM men). Insulin resistance and glycaemic control were associated with a worse lipoprotein profile. Further studies are needed to fully elucidate the implications of all of these advanced lipoprotein profile derangements in the development of CVD events in our, and other, T1DM populations.

## Supplementary information

**Additional file 1: Table S1.** Differences in clinical and laboratory characteristics in study participants according to the study cohort.

**Additional file 2: Table S2.** Differences in clinical and laboratory characteristics in study participants without lipid-lowering drugs.

**Additional file 3: Table S3.** NMR-assessed advanced lipoprotein profile in the control and T1DM groups, and according to cohort study.

**Additional file 4: Table S4.** NMR-assessed advanced lipoprotein profile in control and T1DM groups according to gender in participants without lipid-lowering treatment.

**Additional file 5: Figure S1.** NMR-assessed lipoprotein changes according to quintiles of age. Solid lines represent the control group and dashed lines the T1DM group. Values showed were statin-adjusted. * p<0.05 and ** p<0.01 between-gender differences in the control group. † p<0.05 and †† p<0.01 between-gender differences in the T1DMgroup. Statin use according to quintiles in the control group (men/women): Q1, 0/0%, Q2, 0/2.5%; Q3, 3/3.3%; Q4: 18.2/0%; Q5, 26.3/36.8%. The only statistically difference in the use of statins between genders was in Q4 (p=0.009). Statin use according to quintiles in the T1DM group (men/women): Q1, 10.3/9.8%; Q2, 36.4/32.5%; Q3, 52.5/45.8%; Q4, 56.9/60%; Q5, 68/69.8%. No statistically significant differences in statin use between genders according to quintiles.

**Additional file 6: Table S5.** NMR-assessed advanced lipoprotein profile in T1DM participants according to glycemic control.

**Additional file 7: Figure S2.** Associations between NMR-assessed advanced lipoprotein profile and clinical and laboratory parameters in participants without lipid-lowering drugs. A: Control Group (n = 317); B: T1DM group (n = 275). Solid and open circles indicate positive and negative relationships, respectively. * Available value in n = 191 controls. † Available value in n = 185 controls. ACR: albumin-to-creatinine ratio; ALAT: alanine aminotransferase: BMI: body mass index; eGFR: estimated glomerular filtration rate; FLI: fatty liver index; HDL: high-density lipoprotein; HDL-C: cholesterol content in HDL; HDL-P: HDL particles; HDL-TG: triglyceride content in HDL; LDL: low-density lipoprotein; LDL-C: cholesterol content in LDL; LDL-P: LDL particles; LDL-TG: triglyceride content in LDL; NMR: nuclear magnetic resonance; SBP: systolic blood pressure; VLDL: very low-density lipoprotein; VLDL-C: cholesterol content in VLDL; VLDL-P: VLDL particles; VLDL-TG: triglyceride content in VLDL; WBC: white blood cells; WC: waist circumference.

## Data Availability

The datasets used and/or analysed during the current study are available from the corresponding author on reasonable request and with permission of (didacmauricio@gmail.com) because they contain identifying human information and are unsuitable for public deposition.

## Abbreviations

BMI: Body mass index
CKD-EPI: Chronic Kidney Disease-Epidemiology Collaboration equation
CVD: Cardiovascular disease
FLI: Fatty liver index
HDL: High density lipoprotein
LDL: Low density lipoprotein
HDL: High-density lipoprotein
HDL-C: Cholesterol content in HDL
HDL-P: HDL particles
HDL-TG: Triglyceride content in HDL
LDL: Low-density lipoprotein
hsCRP: High sensitivity C-reactive protein
LDL-C: Cholesterol content in LDL
LDL-P: LDL particles
LDL-TG: Triglyceride content in LDL
NMR: Nuclear magnetic resonance
T1DM: Type 1 diabetes mellitus
VLDL: Very low-density lipoprotein
VLDL-C: Cholesterol content in VLDL
VLDL-P: VLDL particles
VLDL-TG: Triglyceride content in VLDL
