# Supplementary material for: Advanced lipoprotein profile disturbances in type 1 diabetes mellitus: a focus on LDL particles
Source: Cardiovasc Diabetol. 2020 Aug 9;19:126. doi: 10.1186/s12933-020-01099-0 (PMC7416413; doi:10.1186/s12933-020-01099-0)
Supplement: Supplementary file 1 — Additional file 1: Table S1. Differences in clinical and laboratory characteristics in study participants according to the study cohort. [file 12933_2020_1099_MOESM1_ESM.docx]

**Table S1**. Differences in clinical and laboratory characteristics in study participants according to the study cohort.

|  | **Controls**  **(n=347)** | **T1DM-UHGTiP & UHAV**  **(n=319)** | **T1DM-Hospital Clinic**  **(n=189)** | ***p* value**  **(between T1DM cohorts)** | ***p* value**  **(for all)** |
| --- | --- | --- | --- | --- | --- |
| **Clinical characteristics** | | | | | |
| Gender (male) | 151 (43.5) | 152 (47.6) | 109 (57.7) | 0.029 | 0.007 |
| Age (years) | 44.0 (37.0-52.0) | 46.0 (38.0-53.0) | 47.0 (40.8-55.1) | 0.129 | 0.006 |
| Never smokers | 160 (46.1) | 153 (48.0) | 95 (50.3) | 0.616 | 0.651 |
| Hypertension | 32 (9.2) | 92 (28.8) | 58 (30.7) | 0.659 | <0.001 |
| SBP (mmHg) | 120 (110-130) | 128 (115-139) | 126 (117-137) | 0.428 | <0.001 |
| DBP (mmHg) | 75 (70-81) | 75 (68-80) | 81 (74-86) | <0.001 | <0.001 |
| BMI (kg/m^2^)    Obesity (BMI ≥30 kg/m^2^)* | 24.9 (22.8-27.8)  42 (12.3) | 25.6 (22.8-28.2)  56 (17.6) | 26.0 (23.5-28.6)  29 (15.3) | 0.282  0.519 | 0.038  0.162 |
| Waist circumference (cm)  Central obesity ^†^ | 90 (82-99)  127 (37.7) | 89 (80-98)  106 (34.6) | 91 (84-100)  65 (34.8) | 0.039  0.979 | 0.098  0.676 |
| Diabetes duration (years) | --- | 20.0 (14.0-29.0) | 26.5 (20.6-33.4) | <0.001 | --- |
| Diabetic nephropathy^‡^ | --- | 22 (7.0) | 19 (10.1) | 0.218 | --- |
| Diabetic retinopathy^§^ | --- | 128 (41.4) | 72 (38.1) | 0.462 | --- |
| Statin use | 28 (8.1) | 138 (43.3) | 94 (49.7) | 0.157 | <0.001 |
| **Conventional lipid profile (mg/dL)** | | | | | |
| Total cholesterol (mg/dL) | 193 (172-219) | 176 (160-200) | 181 (163-206) | 0.112 | <0.001 |
| HDL-cholesterol  Low HDL-cholesterol^\|\|^ | 58 (49-68)  45 (13.0) | 61 (52-73)  24 (7.6) | 56 (48-69)  26 (13.8) | 0.002  0.025 | 0.001  0.038 |
| LDL-cholesterol  LDL-cholesterol <100  LDL-cholesterol <70 | 116 (96-137)  100 (29.1)  19 (5.5) | 99 (82-116)  159 (50.3)  25 (7.9) | 109 (90-125)  67 (35.4)  11 (5.8) | 0.001  0.001  0.377 | <0.001  <0.001  0.422 |
| Triglycerides  Triglycerides ≥150 | 85 (63-118)  53 (15.4) | 68 (53-88)  17 (5.4) | 72 (56-99)  16 (8.5) | 0.030  0.171 | <0.001  <0.001 |
| Non-HDL cholesterol | 136 (113-160) | 112 (97-132) | 125 (105-142) | <0.001 | <0.001 |
| Remnant cholesterol | 17 (13-23) | 14 (11-18) | 14 (11-19) | 0.039 | <0.001 |
| **Other laboratory characteristics** | | | | | |
| Fasting plasma glucose (mg/dL) | 87 (82-94) | 154 (109-211) | 146 (105-195) | 0.242 | <0.001 |
| Haemoglobin A1c (%) | 5.4 (5.1-5.6) | 7.5 (7.0-8.2) | 7.3 (7.0-7.9) | 0.057 | <0.001 |
| Serum creatinine (mg/dL) | 0.77 (0.67-0.89) | 0.77 (0.65-0.88) | 0.83 (0.73-0.94) | <0.001 | <0.001 |
| eGFR (CKD-EPI; ml/min/1.73m^2^) | 103 (90-111) | 104 (92-112) | 97 (86-105) | <0.001 | <0.001 |
| Alanine aminotransferase | 17 (13-23) | 17 (14-23) | 20 (16-28) | <0.001 | <0.001 |
| γ-glutamyl transpeptidase | 17 (12-25) | 16 (12-22) | 18 (13-28) | 0.002 | 0.008 |
| Leukocyte count (per mm^3^) | 6100 (5000-7307) | 6190 (5100-7600) | 6610 (5550-8380) | 0.001 | <0.001 |
| hsCRP (mg/L) ^¶^ | 1.13 (0.50-2.03 | 1.29 (0.60-2.64) | 1.25 (0.50-3.10) | 0.866 | 0.228 |
| Albumin-to-creatinine ratio (mg/g) | 2.8 (1.4-5.0) | 3.9 (2.0-6.9) | 4.0 (2.0-7.0) | 0.670 | <0.001 |
| Fatty liver index**    Fatty liver index >60 | 23.5 (10.1-54.8)  67 (19.3) | 19.9 (8.2-40.7)  41 (13.7) | 30.1 (12.9-57.5)  30 (22.6) | <0.001  0.022 | 0.001  0.038 |

Data are shown as n (percentage), mean± standard deviation or median (Q1-Q3).

*p* values for group comparisons are reported

BMI: Body Mass Index; DBP: diastolic blood pressure; CSII: continuous subcutaneous insulin insfusion; eGFR: estimated glomerular filtration rate; HDL: high density lipoprotein; hsCRP: high sensitivity C-reactive protein; LDL: low density lipoprotein; SBP: systolic blood pressure; T1DM: type 1 diabetes mellitus; UHAV: University Hospital Arnau de Vilanova; UHGTiP: University Hospital Germans Trias i Pujol.

*Missing values; n=5, n=0 and n=0.

^†^Defined as ≥88 cm in women and ≥102 cm in men. Missing values, n=10, n=13 and n=2.

^‡^Missing values n=3 and n=0.

^§^Missing values n=10 and n=0.

^||^Defined as HDL-cholesterol <50 in women and <40 mg/dL in men.

^¶^Missing values n=162, n= 26 and n=49.

**Missing values n=13, n=20 and n=56.
