# Supplementary material for: Advanced lipoprotein profile disturbances in type 1 diabetes mellitus: a focus on LDL particles
Source: Cardiovasc Diabetol. 2020 Aug 9;19:126. doi: 10.1186/s12933-020-01099-0 (PMC7416413; doi:10.1186/s12933-020-01099-0)
Supplement: Supplementary file 2 — Additional file 2: Table S2. Differences in clinical and laboratory characteristics in study participants without lipid-lowering drugs. [file 12933_2020_1099_MOESM2_ESM.docx]

**Table S2**. Differences in clinical and laboratory characteristics in study participants without lipid-lowering drugs.

|  | **Controls**  **(n=317)** | **T1DM**  **(n=275)** | ***p* value** |
| --- | --- | --- | --- |
| **Clinical characteristics** | | | |
| Gender (male) | 139 (43.8) | 138 (50.2) | 0.124 |
| Age (years) | 42.5 (36.0-50.0) | 42.0 (35.4-50.0) | 0.492 |
| Never smokers | 144 (45.7) | 144 (52.4) | 0.092 |
| Hypertension | 23 (7.3) | 47 (17.1) | <0.001 |
| SBP (mmHg) | 119 (110-129) | 123 (112-134) | 0.003 |
| DBP (mmHg) | 75 (69-81) | 77 (70-83) | 0.164 |
| BMI (kg/m^2^)  Obesity (BMI ≥30 kg/m^2^)* | 24.7 (22.6-27.5)  37 (11.9) | 24.8 (22.3-27.7)  40 (14.5) | 0.775  0.336 |
| Waist circumference (cm)  Central obesity ^†^ | 90 (81-99)  108 (35.1) | 87 (79-98)  73 (27.5) | 0.029  0.054 |
| Diabetes duration (years) | --- | 22.0 (15.0-30.0) | --- |
| Diabetic nephropathy | --- | 15 (5.5) | --- |
| Diabetic retinopathy^‡^ | --- | 96 (35.3) | --- |
|  |  |  |  |
| **Conventional lipid profile** | | | |
| Total cholesterol (mg/dL) | 192 (172-217) | 185 (167-206) | 0.002 |
| HDL-cholesterol (mg/dL)  Low HDL-cholesterol (mg/dL)^§^ | 58 (49-68)  41 (13.0) | 60 (51-73)  31 (11.3) | 0.065  0.529 |
| LDL-cholesterol (mg/dL)  LDL-cholesterol <100 mg/dL  LDL-cholesterol <70 mg/dL | 116 (96-136)  92 (29.2)  17 (5.4) | 109 (92-125)  93 (33.9)  13 (4.7) | 0.004  0.217  0.719 |
| Triglycerides (mg/dL)  Triglycerides ≥150 mg/dL | 84 (62-114)  46 (14.6) | 67 (53-88)  14 (5.1) | <0.001  <0.001 |
| Non-HDL cholesterol (mg/dL) | 135 (113-157) | 125 (105-141) | <0.001 |
| Remnant cholesterol (mg/dL) | 17 (12-22) | 13 (10-18) | <0.001 |
|  | | | |
| **Other laboratory characteristics** | | | |
| Fasting plasma glucose (mg/dL) | 87 (82-93) | 147 (105-199) | <0.001 |
| Haemoglobin A1c (%) | 5.4 (5.1-5.6) | 7.4 (7.0-8.2) | <0.001 |
| Serum creatinine (mg/dL) | 0.77 (0.66-0.89) | 0.78 (0.68-0.90) | 0.608 |
| eGFR (CKD-EPI; ml/min/1.73m^2^) | 103 (93-112) | 104 (94-113) | 0.280 |
| Alanine aminotransferase | 17 (13-23) | 16 (14-22) | 0.911 |
| γ-glutamyl transpeptidase | 16 (12-25) | 15 (12-20) | 0.055 |
| Leukocyte count (per mm^3^) | 6095 (5000-7300) | 6060 (5010-7600) | 0.573 |
| hsCRP (mg/dL) ^\|\|^ | 1.13 (0.50-2.01) | 1.10 (0.50-2.50) | 0.673 |
| Albumin-to-creatinine ratio (mg/g)^¶^ | 2.8 (1.5-4.9) | 3.6 (1.9-6.0) | 0.016 |
| Fatty liver index**  Fatty liver index >60 | 21.2 (9.4-51.6)  57 (18.7) | 17.3 (6.7-40.3)  29 (12.3) | 0.012  0.046 |

Data are shown as n (percentage), mean± standard deviation or median (Q1-Q3).

*p* values for group comparisons are reported

BMI: Body Mass Index; DBP: diastolic blood pressure; eGFR: estimated glomerular filtration rate; HDL: high density lipoprotein; hsCRP: high sensitivity C-reactive protein; LDL: low density lipoprotein; SBP: systolic blood pressure; T1DM: type 1 diabetes mellitus

*Missing values; n=5 and n=0.

^†^Defined as ≥88 cm in women and ≥102 cm in men. Missing values, n=9 and n=10.

^‡^Missing values n=3.

^§^Defined as HDL-cholesterol <50 in women and <40 mg/dL in men.

^||^Missing values n=147 and n=34.

^¶^Data not available for the patients from Mollerusa cohort (n=143).

**Missing values n=12 and n=40.
