# Supplementary material for: Advanced lipoprotein profile disturbances in type 1 diabetes mellitus: a focus on LDL particles
Source: Cardiovasc Diabetol. 2020 Aug 9;19:126. doi: 10.1186/s12933-020-01099-0 (PMC7416413; doi:10.1186/s12933-020-01099-0)
Supplement: Supplementary file 3 — Additional file 3: Table S3. NMR-assessed advanced lipoprotein profile in the control and T1DM groups, and according to cohort study. [file 12933_2020_1099_MOESM3_ESM.docx]

**Table S3:** NMR-assessed advanced lipoprotein profile in the control and T1DM groups, and according to cohort study.

| **NMR variable** | **T1DM-UHGTiP & UHAV (T1DM-1)**  **(n=319)** | **T1DM-Hospital Clinic (T1DM-2)**  **(n=189)** | ***p* value**  **(T1DM cohorts)** | **Sex-adjusted *p* value*** | **Controls**  **(n=347)** | ***p* value**  **(T1DM-1 vs. Controls)** | **Adjusted *p* value^†^** | ***p* value**  **(T1DM-2 vs. Controls)** | **Adjusted *p* value^‡^** |
| --- | --- | --- | --- | --- | --- | --- | --- | --- | --- |
| **VLDL-P number (nmol/L)**  Total  Large  Medium  Small  Ratio Large / Total | 26.6 (21.5-35.6)  0.76 (0.58-0.99)  2.65 (1.82-4.05)  23.1 (18.9-30.3)  0.027 (0.025-0.029) | 31.4 (25.8-42.0)  0.95 (0.71-1.20)  3.17 (2.12-4.66)  27.3 (23.3-34.8)  0.028 (0.025-0.030) | <0.001  <0.001  0.010  <0.001  0.034 | <0.001  <0.001  0.043  <0.001  0.026 | 36.5 (25.5-53.3)  0.93 (0.71-1.24)  3.92 (2.62-5.50)  31.4 (22.1-47.0)  0.025 (0.023-0.027) | <0.001  <0.001  <0.001  <0.001  <0.001 | <0.001  <0.001  <0.001  <0.001  <0.001 | 0.027  0.921  0.001  0.038  <0.001 | <0.001  0.018  <0.001  <0.001  <0.001 |
| **VLDL-P composition (mg/dL)**  VLDL-C  VLDL-TG  Ratio VLDL-C / VLDL-TG | 6.46 (3.47-10.0)  38.0 (30.2-49.8)  0.16 (0.11-0.22) | 9.46 (7.04-13.76)  43.3 (35.8-58.5)  0.21 (0.18-0.24) | <0.001  <0.001  <0.001 | <0.001  0.001  <0.001 | 9.94 (5.44-16.11)  52.8 (36.5-75.3)  0.17 (0.13-0.21) | <0.001  <0.001  0.209 | <0.001  <0.001  0.274 | 0.241  0.001  <0.001 | 0.866  <0.001  <0.001 |
| **VLDL-P size (nm)** | 42.2 (42.0-42.3) | 42.0 (41.8-42.3) | 0.001 | 0.006 | 42.1 (42.0-42.3) | 0.402 | 0.376 | 0.002 | 0.061 |
| **LDL-P number (nmol/L)**  Total  Large  Medium  Small  Ratio Small / Total | 1242.9 (1122.6-1384.3)  178.0 (161.1-198.9)  401.3 (329.5-471.7)  667.5 (610.8-733.1)  0.53 (0.50-0.57) | 1249.1 (1134.6-1380.1)  172.3 (155.1-190.0)  350.9 (277.8-421.3)  715.0 (658.6-772.0)  0.58 (0.54-0.62) | 0.900  0.015  <0.001  <0.001  <0.001 | 0.599  0.032  <0.001  <0.001  <0.001 | 1356.2 (1159.3-1567.7)  191.9 (167.6-217.6)  437.6 (344.6-531.3)  708.6 (623.5-827.3)  0.53 (0.49-0.58) | <0.001  <0.001  <0.001  <0.001  0.745 | <0.001  0.012  0.041  <0.001  0.277 | <0.001  <0.001  <0.001  0.983  <0.001 | <0.001  <0.001  <0.001  0.152  <0.001 |
| **LDL-P composition (mg/dL)**  LDL-C (mg/dL)  LDL-TG (mg/dL)  Ratio LDL-C / LDL-TG | 121.2 (109.9-136.9)  15.5 (13.1-18.3)  8.01 (7.08-9.02) | 123.3 (111.2-137.2)  13.3 (11.9-15.8)  9.20 (8.03-10.16) | 0.676  <0.001  <0.001 | 0.922  <0.001  <0.001 | 132.9 (113.5-154.2)  16.6 (13.4-20.4)  8.20 (7.25-9.13) | <0.001  0.005  0.132 | <0.001  0.020  0.889 | <0.001  <0.001  <0.001 | 0.004  <0.001  <0.001 |
| **LDL-P size (nm)** | 21.0±0.24 | 21.0±0.31 | 0.029 | 0.045 | 21.0±0.27 | 0.841 | 0.053 | 0.036 | 0.610 |
| **HDL-P number (μmol/L)**  Total  Large  Medium  Small  Ratio Small / Total | 32.5 (28.8-36.5)  0.28 (0.25-0.32)  10.54 (8.96-12.38)  21.4 (18.8-24.2)  0.66 (0.63-0.69) | 30.6 (27.8-34.7)  0.27 (0.25-0.29)  10.42 (9.31-11.74)  20.0 (18.1-23.1)  0.65 (0.63-0.68) | 0.009  0.004  0.572  0.006  0.081 | 0.160  0.033  0.602  0.049  0.021 | 29.0 (26.0-33.2)  0.26 (0.23-0.30)  9.12 (7.90-10.60)  19.9 (17.4-22.8)  0.67 (0.64-0.71) | <0.001  <0.001  <0.001  <0.001  <0.001 | <0.001  <0.001  <0.001  0.001  <0.001 | 0.001  0.422  <0.001  0.344  <0.001 | <0.001  0.086  <0.001  0.166  <0.001 |
| **HDL-P composition (mg/dL)**  HDL-C (mg/dL)  HDL-TG (mg/dL)  Ratio HDL-C / HDL-TG | 64.0 (56.3-74.0)  13.6 (11.5-16.3)  4.66 (3.91-5.70) | 60.7 (53.6-70.2)  16.5 (14.1-19.5)  3.79 (3.17-4.46) | 0.030  <0.001  <0.001 | 0.370  <0.001  <0.001 | 56.2 (49.2-65.5)  12.7 (10.3-15.7)  4.49 (3.61-5.52) | <0.001  0.001  0.060 | <0.001  0.140  <0.001 | <0.001  <0.001  <0.001 | <0.001  <0.001  0.006 |
| **HDL-P size (nm)** | 8.23±0.06 | 8.27±0.07 | <0.001 | <0.001 | 8.21±0.06 | <0.001 | <0.001 | <0.001 | <0.001 |
| **Other atherogenic variables**  Non-HDL-P (nmol/L)  Ratio LDL-P / HDL-P  Ratio total-P / HDL-P | 1241.4 (1127.2-1387.2)  38.5 (33.1-44.8)  39.6 (33.8-45.9) | 1238.2 (1132.9-1374.1)  40.2 (34.6-48.3)  41.7 (34.6-48.3) | 0.856  0.143  0.092 | 0.864  0.368  0.487 | 1377.2 (1170.7-1578.5)  46.1 (37.2-56.7)  47.7 (38.3-58.4) | <0.001  <0.001  <0.001 | <0.001  <0.001  <0.001 | <0.001  <0.001  <0.001 | <0.001  <0.001  <0.001 |

Data are shown as median (Q1-Q3) or mean ± standard deviation.

*Sex-adjusted differences between the two T1DM cohorts.

^†^Differences between T1DM from Hospital Universitari Gernans Trias I Pujol & Hospital Universitari Arnau de Vilanova and controls adjusted for age, sex, body mass index, leukocyte count and statin use.

^‡^Differences between T1DM from Hospital Clínic and controls adjusted for age, sex, body mass index, leucocyte count and statin use.

HDL: high-density lipoprotein; HDL-C: cholesterol content in HDL; HDL-P: HDL particles; HDL-TG: triglyceride content in HDL; LDL low-density lipoprotein; LDL-C: cholesterol content in LDL; LDL-P: LDL particles; LDL-TG: triglyceride content in LDL; NMR: nuclear magnetic resonance; T1DM: type 1 diabetes mellitus; UHAV: University Hospital Arnau de Vilanova; UHGTiP: University Hospital Germans Trias i Pujol; VLDL: very low-density lipoprotein; VLDL-C: cholesterol content in VLDL; VLDL-P: VLDL particles; VLDL-TG: triglyceride content in VLDL.
