# Supplementary material for: Advanced lipoprotein profile disturbances in type 1 diabetes mellitus: a focus on LDL particles
Source: Cardiovasc Diabetol. 2020 Aug 9;19:126. doi: 10.1186/s12933-020-01099-0 (PMC7416413; doi:10.1186/s12933-020-01099-0)
Supplement: Supplementary file 4 — Additional file 4: Table S4. NMR-assessed advanced lipoprotein profile in control and T1DM groups according to gender in participants without lipid-lowering treatment. [file 12933_2020_1099_MOESM4_ESM.docx]

**Table S4**. NMR-assessed advanced lipoprotein profile in control and T1DM groups according to gender in participants without lipid-lowering treatment.

|  | **Control (n=317)** | | | **T1DM (n=275)** | | | **Men (control vs. T1DM)** | **Women (control vs. T1DM)** |
| --- | --- | --- | --- | --- | --- | --- | --- | --- |
| **NMR variable** | **Men** | **Women** | **p** | **Men** | **Women** | **p** |  |  |
| **VLDL-P number (nmol/L)**  Total  Large  Medium  Small  Ratio Large / Total | 41.0 (31.2-67.5)  1.05 (0.81-1.50)  4.35 (2.93-6.21)  35.9 (27.6-59.6)  0.024 (0.023-0.027) | 31.8 (23.6-45.0)  0.86 (0.60-1.10)  3.32 (2.33-4.96)  27.5 (20.0-39.2)  0.026 (0.023-0.028) | <0.001  <0.001  <0.001  <0.001  <0.001 | 30.8 (23.5-43.9)  0.89 (0.70-1.21)  3.38 (2.33-4.75)  26.3 (20.2-37.8)  0.028 (0.026-0.030) | 24.9 (20.5-31.2)  0.70 (0.55-0.93)  2.42 (1.61-3.64)  21.6 (18.5-27.3)  0.027 (0.025-0.029) | <0.001  <0.001  <0.001  <0.001  0.017 | <0.001  0.003  <0.001  <0.001  <0.001 | <0.001  <0.001  <0.001  <0.001  0.001 |
| **VLDL-P composition (mg/dL)**  VLDL-C  VLDL-TG  Ratio VLDL-C / VLDL-TG | 11.15 (6.19-18.90)  59.2 (43.8-93.4)  0.17 (0.13-0.21) | 7.64 (4.37-12.57)  45.3 (32.9-63.1)  0.17 (0.13-0.20) | <0.001  <0.001  0.969 | 9.00 (5.51-14.80)  42.4 (33.2-62.3)  0.20 (0.15-0.24) | 6.38 (3.47-9.41)  35.2 (28.7-44.3)  0.17 (0.12-0.22) | <0.001  <0.001  0.013 | 0.032  <0.001  0.002 | 0.004  <0.001  0.565 |
| **VLDL-P size (nm)** | 42.1 (42.0-42.2) | 42.2 (42.0-42.3) | 0.046 | 42.2 (42.0-42.4) | 42.1 (41.9-42.3) | 0.011 | 0.008 | 0.029 |
| **LDL-P number (nmol/L)**  Total  Large  Medium  Small  Ratio Small / Total | 1409.0 (1203.8-1602.3)  191.4 (166.0-217.6)  430.1 (323.7-531.3)  773.5 (692.9-873.6)  0.56 (0.52-0.59) | 1309.9 (1129.0-1491.3)  192.2 (169.3-214.0)  435.8 (352.3-519.6)  661.9 (588.6-749.8)  0.51 (0.48-0.55) | 0.002  1.000  0.614  <0.001  <0.001 | 1285.7 (1168.1-1396.7)  182.3 (167.8-195.7)  389.7 (330.9-454.9)  700.8 (647.2-772.0)  0.56 (0.51-0.58) | 1249.7 (1138.0-1407.6)  181.0 (164.0-205.8)  392.9 (332.3-494.7)  669.9 (613.9-743.6)  0.53 (0.49-0.57) | 0.341  0.918  0.220  0.009  0.003 | <0.001  0.036  0.008  <0.001  0.309 | 0.168  0.033  0.024  0.378  0.008 |
| **LDL-P composition (mg/dL)**  LDL-C (mg/dL)  LDL-TG (mg/dL)  Ratio LDL-C / LDL-TG | 136.8 (116.9-157.0)  16.2 (13.0-20.1)  8.55 (7.63-9.58) | 130.3 (111.1-148.5)  16.5 (13.7-20.5)  7.98 (7.06-8.99) | 0.035  0.741  0.003 | 127.6 (116.6-138.3)  14.1 (12.4-16.8)  8.97 (7.76-10.09) | 124.7 (113.4-139.2)  15.1 (12.3-18.6)  8.23 (7.20-9.84) | 0.448  0.139  0.019 | 0.003  0.001  0.078 | 0.189  0.019  0.048 |
| **LDL-P size (nm)** | 20.9±0.26 | 21.1±0.23 | <0.001 | 21.0±0.22 | 21.1±0.26 | 0.031 | <0.001 | 0.109 |
| **HDL-P number (μmol/L)**  Total  Large  Medium  Small  Ratio Small / Total | 27.0 (23.9-29.7)  0.25 (0.22-0.28)  8.01 (7.13-9.13)  18.8 (16.0-21.0)  0.69 (0.65-0.71) | 30.9 (27.6-34.9)  0.28 (0.25-0.31)  10.00 (8.80-11.54)  20.9 (18.2-23.5)  0.66 (0.64-0.70) | <0.001  <0.001  <0.001  <0.001  <0.001 | 29.3 (25.5-33.3)  0.26 (0.24-0.29)  9.61 (8.57-10.73)  19.4 (16.7-22.5)  0.66 (0.63-0.69) | 33.5 (29.2-37.6)  0.29 (0.26-0.33)  11.71 (9.93-13.72)  21.2 (19.1-23.9)  0.64 (0.62-0.67) | <0.001  <0.001  <0.001  <0.001  <0.001 | <0.001  0.010  <0.001  0.051  <0.001 | 0.001  0.001  <0.001  0.259  <0.001 |
| **HDL-P composition (mg/dL)**  HDL-C (mg/dL)  HDL-TG (mg/dL)  Ratio HDL-C / HDL-TG | 51.2 (45.7-57.8)  11.2 (9.0-13.8)  4.76 (3.70-5.72) | 59.7 (53.3-69.6)  13.4 (11.2-16.5)  4.41 (3.58-5.43) | <0.001  <0.001  0.218 | 58.3 (51.6-65.6)  13.0 (10.6-15.7)  4.55 (3.71-5.75) | 68.8 (58.8-79.1)  14.3 (12.2-17.2)  4.75 (3.95-5.68) | <0.001  0.001  0.339 | <0.001  <0.001  0.508 | <0.001  0.060  0.114 |
| **HDL-P size (nm)** | 8.20±0.06 | 8.22±0.05 | <0.001 | 8.24±0.07 | 8.27±0.06 | 0.001 | <0.001 | <0.001 |
| **Other atherogenic variables**  Non-HDL-P (nmol/L)  Ratio LDL-P / HDL-P  Ratio total-P / HDL-P | 1434.8 (1224.5-1634.7)  52.0 (44.0-63.4)  53.9 (45.5-65.5) | 1311.8 (1121.3-1502.2)  40.9 (34.1-50.6)  41.9 (35.2-51.7) | <0.001  <0.001  <0.001 | 1292.7 (1178.2-1412.6)  43.0 (37.5-49.9)  44.4 (38.6-52.1) | 1241.4 (1132.1-1402.0)  37.6 (32.8-43.8)  38.2 (33.5-44.7) | 0.129  <0.001  <0.001 | <0.001  <0.001  <0.001 | 0.087  0.002  0.001 |

Data are shown as median (Q1-Q3) or mean ± standard deviation.

HDL: high-density lipoprotein; HDL-C: cholesterol content in HDL; HDL-P: HDL particles; HDL-TG: triglyceride content in HDL; LDL low-density lipoprotein; LDL-C: cholesterol content in LDL; LDL-P: LDL particles; LDL-TG: triglyceride content in LDL; NMR: nuclear magnetic resonance; T1DM: type 1 diabetes mellitus; VLDL: very low-density lipoprotein; VLDL-C: cholesterol content in VLDL; VLDL-P: VLDL particles; VLDL-TG: triglyceride content in VLDL.
