# Supplementary figures and images for: Advanced lipoprotein profile disturbances in type 1 diabetes mellitus: a focus on LDL particles
Source: Cardiovasc Diabetol. 2020 Aug 9;19:126. doi: 10.1186/s12933-020-01099-0 (PMC7416413; doi:10.1186/s12933-020-01099-0)

## Slide 1
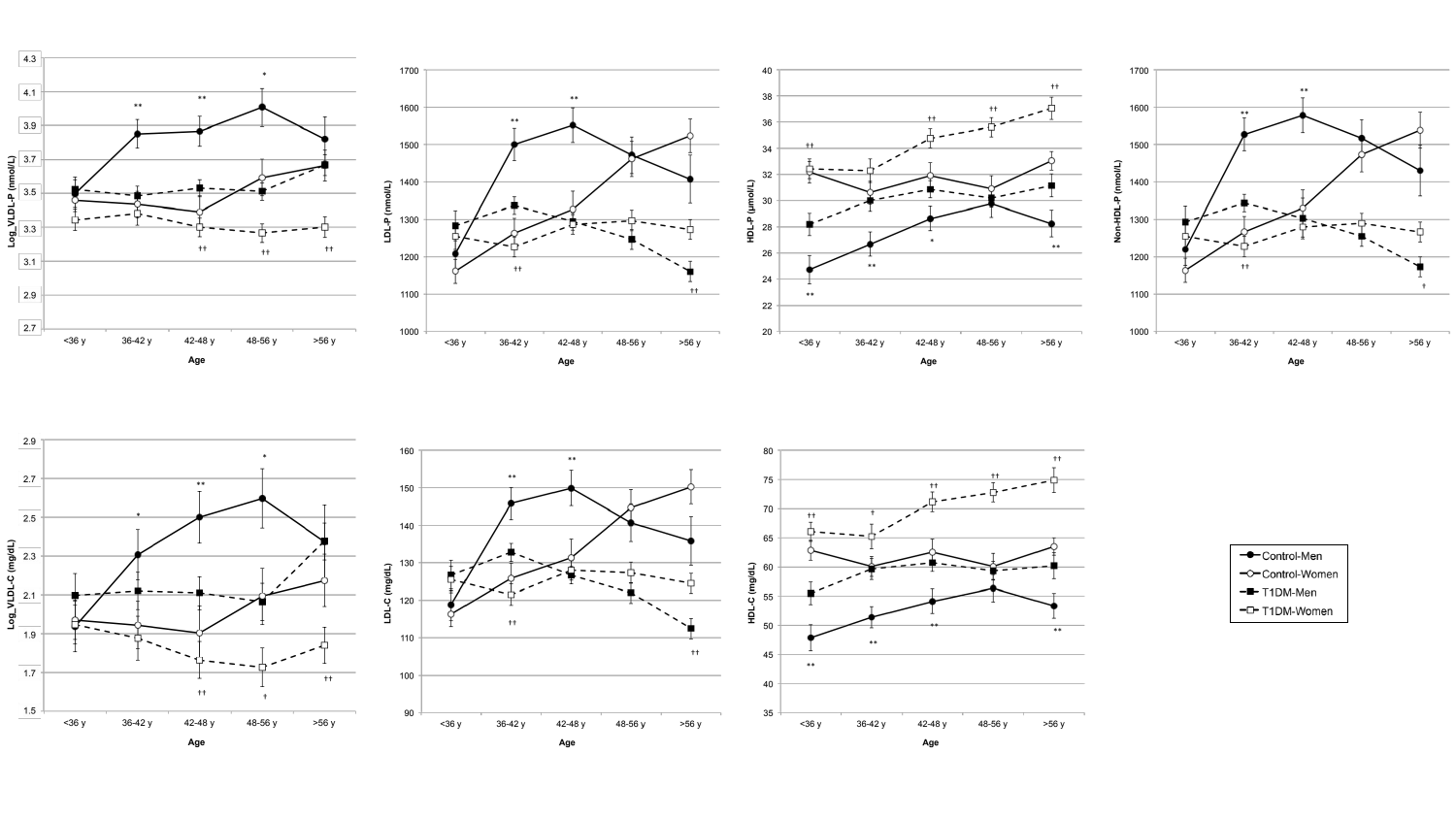

Supplement: Supplementary file 5 — Additional file 5: Figure S1. NMR-assessed lipoprotein changes according to quintiles of age. Solid lines represent the control group and dashed lines the T1DM group. Values showed were statin-adjusted. * p<0.05 and ** p<0.01 between-gender differences in the control group. † p<0.05 and †† p<0.01 between-gender differences in the T1DMgroup. Statin use according to quintiles in the control group (men/women): Q1, 0/0%, Q2, 0/2.5%; Q3, 3/3.3%; Q4: 18.2/0%; Q5, 26.3/36.8%. The only statistically difference in the use of statins between genders was in Q4 (p=0.009). Statin use according to quintiles in the T1DM group (men/women): Q1, 10.3/9.8%; Q2, 36.4/32.5%; Q3, 52.5/45.8%; Q4, 56.9/60%; Q5, 68/69.8%. No statistically significant differences in statin use between genders according to quintiles. [file 12933_2020_1099_MOESM5_ESM.pptx]

## Slide 1
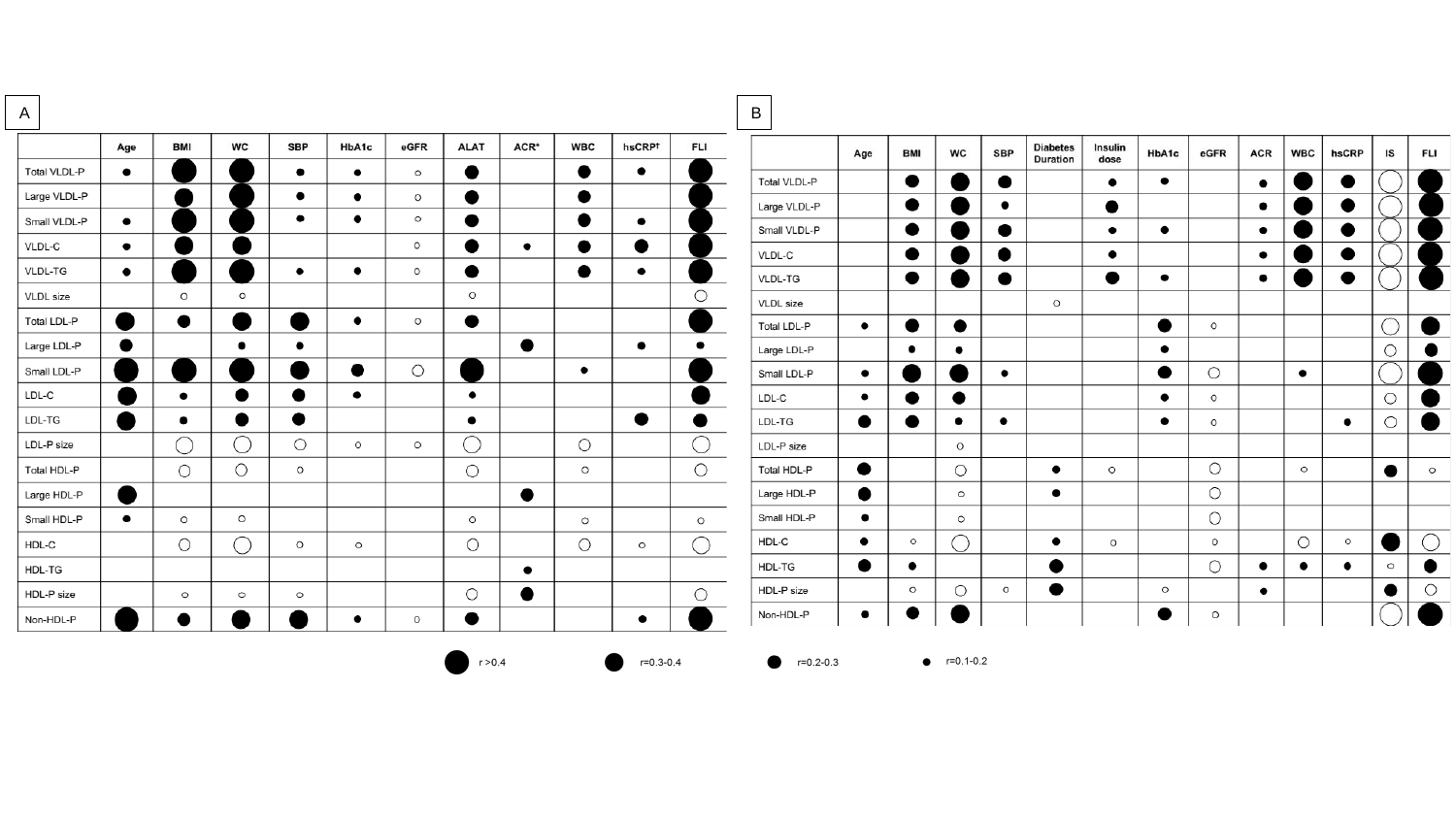

A
B

Supplement: Supplementary file 7 — Additional file 7: Figure S2. Associations between NMR-assessed advanced lipoprotein profile and clinical and laboratory parameters in participants without lipid-lowering drugs. A: Control Group (n = 317); B: T1DM group (n = 275). Solid and open circles indicate positive and negative relationships, respectively. * Available value in n = 191 controls. † Available value in n = 185 controls. ACR: albumin-to-creatinine ratio; ALAT: alanine aminotransferase: BMI: body mass index; eGFR: estimated glomerular filtration rate; FLI: fatty liver index; HDL: high-density lipoprotein; HDL-C: cholesterol content in HDL; HDL-P: HDL particles; HDL-TG: triglyceride content in HDL; LDL: low-density lipoprotein; LDL-C: cholesterol content in LDL; LDL-P: LDL particles; LDL-TG: triglyceride content in LDL; NMR: nuclear magnetic resonance; SBP: systolic blood pressure; VLDL: very low-density lipoprotein; VLDL-C: cholesterol content in VLDL; VLDL-P: VLDL particles; VLDL-TG: triglyceride content in VLDL; WBC: white blood cells; WC: waist circumference. [file 12933_2020_1099_MOESM7_ESM.pptx]
