# Supplementary material for: Advanced lipoprotein profile disturbances in type 1 diabetes mellitus: a focus on LDL particles
Source: Cardiovasc Diabetol. 2020 Aug 9;19:126. doi: 10.1186/s12933-020-01099-0 (PMC7416413; doi:10.1186/s12933-020-01099-0)
Supplement: Supplementary file 6 — Additional file 6: Table S5. NMR-assessed advanced lipoprotein profile in T1DM participants according to glycemic control. [file 12933_2020_1099_MOESM6_ESM.docx]

**Table S5.** NMR-assessed advanced lipoprotein profile in T1DM participants according to glycemic control.

| **NMR variable** | **HbA1c <7%**  **(n=149)** | **HbA1c 7-8.5%**  **(n=284)** | **HbA1c >8.5%**  **(n=73)** | ***p* value** | ***p***  **value*** |
| --- | --- | --- | --- | --- | --- |
| **VLDL-P number (nmol/L)**  Total  Large  Medium  Small  Ratio Large / Total | 28.8 (22.5-34.0)  0.82 (0.61-1.00)  2.65 (1.86-3.65)  25.5 (19.6-29.7)  0.028 (0.026-0.030) | 28.8 (22.6-37.7)  0.81 (0.63-1.04)  2.73 (1.83-4.21)  25.3 (20.1-32.1)  0.027 (0.025-0.029) | 31.9 (24.6-49.6)  0.88 (0.69-1.20)  3.62 (2.31-5.94)  27.3 (21.3-42.2)  0.025 (0.024-0.030) | 0.003  0.041  0.005  0.004  <0.001 | 0.010  0.122  0.015  0.014  <0.001 |
| **VLDL-P composition (mg/dL)**  VLDL-C  VLDL-TG  Ratio VLDL-C / VLDL-TG | 7.78 (4.71-10.87)  40.4 (32.0-46.1)  0.19 (0.14-0.24) | 7.37 (4.19-11.46)  40.2 (32.1-53.5)  0.19 (0.13-0.23) | 8.70 (5.44-14.88)  45.9 (35.3-69.5)  0.18 (0.14-0.23) | 0.082  0.001  0.666 | 0.208  0.004  0.468 |
| **VLDL-P size (nm)** | 42.1 (41.9-42.3) | 42.1 (41.9-42.3) | 42.2 (41.9-42.4) | 0.124 | 0.178 |
| **LDL-P number (nmol/L)**  Total  Large  Medium  Small  Ratio Small / Total | 1203.6 (1118.3-1319.3)  175.2 (159.0-190.3)  369.6 (315.8-444.9)  668.8 (601.2-718.3)  0.55 (0.52-0.58) | 1246.3 (1125.1-1371.5)  173.4 (158.8-193.5)  369.0 (314.5-457.2)  682.9 (625.4-755.5)  0.56 (0.51-0.59) | 1366.4 (1197.5-1564.8)  188.3 (167.6-219.9)  417.5 (336.9-516.9)  738.5 (668.4-814.6)  0.55 (0.51-0.59) | <0.001  <0.001  0.005  <0.001  0.760 | <0.001  0.001  0.007  <0.001  0.721 |
| **LDL-P composition (mg/dL)**  LDL-C (mg/dL)  LDL-TG (mg/dL)  Ratio LDL-C / LDL-TG | 119.3 (109.3-133.2)  14.3 (12.2-16.5)  8.55 (7.59-9.71) | 121.9 (109.4-135.8)  14.3 (12.5-17.6)  8.23 (7.32-9.63) | 135.1 (117.9-153.0)  16.3 (13.0-21.0)  8.13 (7.23-9.27) | <0.001  <0.001  0.158 | <0.001  <0.001  0.175 |
| **LDL-P size (nm)** | 21.1±0.24 | 21.0±0.28 | 21.0±0.29 | 0.455 | 0.500 |
| **HDL-P number (μmol/L)**  Total  Large  Medium  Small  Ratio Small / Total | 31.9 (28.3-36.2)  0.28 (0.25-0.31)  10.61 (9.59-12.08)  20.9 (18.0-23.9)  0.65 (0.62-0.68) | 31.7 (28.2-36.0)  0.27 (0.25-0.31)  10.42 (9.25-12.26)  20.8 (18.7-23.6)  0.66 (0.63-0.69) | 31.2 (27.0-35.9)  0.28 (0.25-0.32)  9.75 (8.63-11.37)  21.1 (18.2-24.6)  0.67 (0.64-0.70) | 0.887  0.201  0.016  0.312  <0.001 | 0.686  0.077  0.039  0.149  0.001 |
| **HDL-P composition (mg/dL)**  HDL-C (mg/dL)  HDL-TG (mg/dL)  Ratio HDL-C / HDL-TG | 63.2 (56.2-73.9)  14.5 (12.0-17.6)  4.56 (3.69-5.31) | 62.9 (55.05-72.7)  14.9 (12.4-18.0)  4.29 (3.54-5.31) | 60.1 (52.5-68.8)  14.8 (11.4-16.9)  4.25 (3.59-5.35) | 0.319  0.785  0.650 | 0.695  0.628  0.856 |
| **HDL-P size (nm)** | 8.26±0.07 | 8.24±0.06 | 8.23±0.06 | <0.001 | 0.001 |
| **Other atherogenic variables**  Non-HDL-P (nmol/L)  Ratio LDL-P / HDL-P  Ratio total-P / HDL-P | 1198.9 (1112.3-1325.8)  37.9 (32.8-44.2)  38.7 (33.8-45.0) | 1254.3 (1124.3-1374.2)  39.1 (33.3-44.9)  40.3 (33.8-46.1) | 1387.7 (1198.3-1567.8)  41.7 (34.6-50.5)  43.2 (35.6-51.8) | <0.001  <0.001  <0.001 | <0.001  0.001  0.001 |

Data are shown as median (Q1-Q3) or mean ± standard deviation.

*p-value adjusted for age, sex, statin use, BMI and leukocyte count.

HDL: high-density lipoprotein; HDL-C: cholesterol content in HDL; HDL-P: HDL particles; HDL-TG: triglyceride content in HDL; LDL low-density lipoprotein; LDL-C: cholesterol content in LDL; LDL-P: LDL particles; LDL-TG: triglyceride content in LDL; NMR: nuclear magnetic resonance; T1DM: type 1 diabetes mellitus; VLDL: very low-density lipoprotein; VLDL-C: cholesterol content in VLDL; VLDL-P: VLDL particles; VLDL-TG: triglyceride content in VLDL.
